# Impact of SARS-CoV-2 Pandemic on Patients with Primary Immunodeficiency

**DOI:** 10.1007/s10875-020-00928-x

**Published:** 2020-12-01

**Authors:** Samaneh Delavari, Hassan Abolhassani, Farhad Abolnezhadian, Fateme Babaha, Sara Iranparast, Hamid Ahanchian, Nasrin Moazzen, Mohammad Nabavi, Saba Arshi, Morteza Fallahpour, Mohammad Hassan Bemanian, Sima Shokri, Tooba Momen, Mahnaz Sadeghi-Shabestari, Rasol Molatefi, Afshin Shirkani, Ahmad Vosughimotlagh, Molood Safarirad, Meisam Sharifzadeh, Salar Pashangzadeh, Fereshte Salami, Paniz Shirmast, Arezou Rezaei, Tannaz Moeini Shad, Minoo Mohraz, Nima Rezaei, Lennart Hammarström, Reza Yazdani, Asghar Aghamohamamdi

**Affiliations:** 1 Research Center for Primary Immunodeficiencies, Pediatrics Center of Excellence, Children’s Medical Center, Tehran University of Medical Sciences, Tehran, Iran; 2 Division of Clinical Immunology, Department of Laboratory Medicine, Karolinska Institute at Karolinska University Hospital Huddinge, Stockholm, Sweden; 3 Department of Pediatrics, Abuzar Children’s Hospital, Ahvaz Jundishapur University of Medical Sciences, Ahvaz, Iran; 4 Department of Immunology, Faculty of Medical Sciences, Tarbiat Modares University, Tehran, Iran; 5 Department of immunology, Faculty of Medical Sciences, Ahvaz Jundishapur University of Medical Sciences, Ahvaz, Iran; 6 Allergy Research Center, Mashhad University of Medical Sciences, Mashhad, Iran; 7 Department of Allergy and Clinical Immunology, Rasool e Akram Hospital, Iran University of Medical Sciences, Tehran, Iran; 8 Department of Allergy and Clinical Immunology, Child Growth and Development Research Center, Research Institute for Primordial Prevention of Noncommunicable Disease, Isfahan University of Medical Sciences, Isfahan, Iran; 9 Immunology research center of Tabriz, TB and lung research center of Tabriz, children hospital, Tabriz University of Medical Science, Tabriz, Iran; 10 Department of Pediatrics, Bo-Ali Children’s Hospital of Ardabil University of Medical Sciences, Ardabil, Iran; 11 Allergy and Clinical Immunology Department, Bushehr University of Medical Sciences, School of Medicine, Bushehr, Iran; 12 Department of Pediatrics, North Khorasan University of Medical Sciences, Bojnurd, Iran; 13 Division of Pediatric Critical Care, Children Medical Center, Tehran University of Medical Science, Tehran, Iran; 14 Iranian Research Center for HIV/AIDS, Iranian Institute for Reduction of High-Risk Behaviors, Tehran University of Medical Sciences, Tehran, Iran

**Keywords:** COVID-19, Primary immunodeficiency, Severe viral infection, Mortality rate

## Abstract

Although it is estimated that COVID-19 life-threatening conditions may be diagnosed in less than 1:1000 infected individuals below the age of 50, but the real impact of this pandemic on pediatric patients with different types of primary immunodeficiency (PID) is not elucidated. The current prospective study on a national registry of PID patients showed that with only 1.23 folds higher incidence of infections, these patients present a 10-folds higher mortality rate compared to population mainly in patients with combined immunodeficiency and immune dysregulation. Therefore, further management modalities against COVID-19 should be considered to improve the survival rate in these two PID entities using hematopoietic stem cell transplantation and immunomodulatory agents.

**Supplementary Information:**

The online version contains supplementary material available at 10.1007/s10875-020-00928-x.

## Introduction

The novel coronavirus disease, known as coronavirus disease 2019 (COVID-19), is an acute infectious respiratory disease that first emerged in Wuhan, China, in late 2019 and is characterized as a pandemic in mid-March 2020 by World Health Organization [1]. It has been found that COVID-19 causes severe acute respiratory syndrome (SARS, therefore coined as SARS-CoV-2) similar to two other RNA viruses from the *Coronoviridea* family SARS-CoV-1 and Middle East respiratory syndrome coronavirus (MERS-CoV). SARS-CoV-2 main route of entry to a human host is the angiotensin-converting enzyme 2 (ACE2) receptor. ACE2 receptor is ubiquitously expressed on the surfaces of various cell types, including cells of the airway epithelium which is the major site of infection [2]. Besides the ACE2 receptor, SARS-CoV-2 uses the transmembrane serine protease 2 (TMPRSS2), a cellular serine protease, for the host cell entry, which activates the SARS-CoV-2 spike protein by cleaving the Furin site at the S1/S2 subunits [3, 4]. Upon entry, the virus can subsequently affect endosomes, and eventually, fuse viral and lysosomal membranes [5]. Coronaviruses are pathogens affecting both humans and animals; particularly SARS-CoV-2 is highly contagious and can be transmitted directly through respiratory droplets often even before the infected person shows symptoms or indirectly by touching infected surfaces and following inoculation within mucosal layers [2, 6].

SARS-CoV-2 infection results in the development of an unusual form of pneumonia and can cause acute respiratory distress syndrome (ARDS) [7]. More than 35 million infected cases were diagnosed worldwide, with a mortality rate of 4% (1–20%) and about 20% of those who get COVID-19 become seriously ill and require oxygen, with 5% becoming critically ill and needing intensive care (https://www.who.int/emergencies/diseases/novel-coronavirus-2019/question-and-answers-hub/q-a-detail/coronavirus-disease-covid-19), mainly in old patients with multiple comorbidities. Of note, it is estimated that life-threatening conditions may be observed in less than 1:1000 infected individuals below the age of 50 [7], suggesting some underlying susceptibility factors in this selected group of patients. Hence, it is expected to see severe COVID-19 cases in individuals with primary immunodeficiencies (PIDs). Due to poor cellular immunity and viral control, disease severity is expected to be higher in combined immunodeficiencies than patients with humoral defects. Patients with immune dysregulation may also be vulnerable to the disease because of the risk of an adverse unregulated inflammatory response in these patients. Noteworthy, patients with defects in their innate immune system, especially those with defects in the interferon (IFN) pathway, seem to face more frequently a critical course of the disease [8]. PID prevalence (range 1:8500–1:100000 for symptomatic patients) and the proportion of PID entities (more than 400 heterogeneous defects) in each country may impact the number of patients at risk due to COVID-19. Recent discoveries identified the role of defective immune-related genes involved in RNA-virus infection susceptibilities with a critical function in IFN pathways including IFN receptors (*IFNAR1* and *IFNAR2*), signal transducer and activator of transcription of IFN (*STAT1* and *STAT2*), IFN regulatory factors (*IRF7*, *IRF9*, and *IFIH1*), and RNA polymerase III (*POLR3A, POLR3C*, and *POLR3F*) [9].

To investigate this hypothesis, we conducted a prospective study to compare the rate and outcomes of COVID-19 infection between diagnosed cases in the Iranian PID registry with population-based data.

## Methods

### Iranian Primary Immunodeficiency Registry (IPIDR)

This study was conducted as a cohort of patients, prospectively enrolled in the IPIDR from the “National PID Network” [10]. The IPIDR is managed by the Research Center for Immunodeficiencies (Tehran, Iran) and its main aim is to provide epidemiological, clinical, and molecular data of PID in Iran. By the latest estimation in 2020, Iran has a population of 84,012,442 citizens (with an average annual birth rate of 1,300,000) and according to the age structure, 23.7% are less than 14 years old. Data on COVID-19 infection in PID patients were compared with the normal population (471,772 patients, incidence of 2036 cases per day, 26,957 total death, https://www.worldometers.info/coronavirus/, reported from Iranian Ministry of Health, Tehran, Iran, access data 4 October 2020). This study received approval from the Ethics Committee of the Tehran University of Medical Science. Moreover, written informed consent has been obtained from all patients, their parents, or legal guardians.

The registry database is hosted in the Children’s Medical Center (Tehran, Iran) which serves as a referral hospital for suspected or diagnosed PID cases. Moreover, 38 medical centers, affiliated to 27 medical science universities, collaborated in the registry program from the major provinces of the country to form the PID network. All participating centers had access to national guidelines [11] and necessary laboratory equipment for clinical and immunological evaluations [10]. Subsequently, cases with a suspected diagnosis were referred and re-evaluated in the Children’s Medical Center for a definitive diagnosis.

### Clinical and Immunologic Diagnoses in COVID-19-Infected PID Patients

The clinical diagnosis of the PID patients was made according to the criteria of the European Society for immunodeficiencies (ESID) [12]. A questionnaire surveyed the patients’ demographic information, age of disease onset, age of diagnosis, family history, a detailed clinical history that included vaccination history and associated adverse reactions, recurrent infections, physical examination findings, laboratory data, and treatment history. Secondary defects of the immune system, including those caused by human immunodeficiency virus (HIV) were ruled out. Laboratory evaluations were performed in the study group as indicated, including complete blood and differential counts, serum protein profile and immunoglobulin levels, serum IgG subclass levels, isohemagglutinin titers, specific antibody responses, disease-specific autoantibody measurements, flow cytometric evaluation of lymphocyte subsets, nitro blue tetrazolium dye/dihydrorhodamine test, granulocyte function and chemotaxis tests, lymphocyte transformation and T cell function tests, radiosensitivity, measurement of complement component levels, and hemolytic complement activity [10]. Microbiological, pathological and imaging evaluations were performed for clinical diagnosis when required. A computerized database program (new registry section in http://rcid.tums.ac.ir/, access data 4 October 2020) was implemented for data entry. After reviewing the cases by the administrator of the system for duplicated cases, patients with incomplete diagnostic criteria were excluded. The online database was updated frequently for approved patients and all follow-up data sent by the end of the study period were included. If the diagnosis of PID were confirmed before the time of COVID-19 patients, following evaluation were performed including new clinical presentation, high-resolution computed tomography (HRCT), and reverse transcriptase-polymerase chain reactions (RT-PCR) as well as complete blood count (CBC), C-reactive protein (CRP), and erythrocyte sedimentation rate (ESR). For patients with a recent diagnosis of PID, a comprehensive questionnaire was provided and filled out for each patient which consists of demographical data, clinical manifestations related to their PID and COVID-19, laboratory tests findings such covering both PID and COVID-19 diagnoses confirmation. Furthermore, the type of treatment and outcome were also provided for each patient.

### Genetic Analysis and Diagnoses in COVID-19-Infected PID Patients

Genomic DNA was extracted from whole blood from patients who agreed with genetic tests and for patients with classical clinical presentations suggestive of a specific PID, targeted sequencing was performed (Table S1). For patients in whom targeted sequencing failed or who had a clinical presentation resembling several genetic defects, whole-exome sequencing was performed to detect single nucleotide variants, insertion/deletions, and large deletions using a pipeline described previously [13, 14]. Candidate variants were evaluated by the Combined Annotation Dependent Depletion (CADD) algorithm and an individual gene cutoff given by using the Mutation Significance Cutoff (MSC) was considered for impact predictions [15]. The Gene Damage Index (GDI) server and the Human Gene Connectome (HGC) were used to making a combined effect prediction [15]. The pathogenicity of all disease attributable gene variants was re-evaluated using the updated guideline for interpretation of molecular sequencing by the American College of Medical Genetics and Genomics criteria [16, 17], considering the allele frequency in the population database, computational data, immunological data, familial segregation and parental data (confirmatory Sanger sequencing for probands and their parents), and clinical phenotyping.

### Classification of COVID-19-Infected PID Patients

After confirmation of their clinical and genetic diagnosis, patients were classified according to the International Union of Immunological Societies (IUIS) updated classification including 9 categories of immunodeficiencies affecting cellular and humoral immunity (non-syndromic combined immunodeficiency or CID), combined immunodeficiencies with associated or syndromic features (syndromic CID), predominantly antibody deficiencies (PAD), diseases of immune dysregulation, congenital defects of phagocyte number or function (phagocytic disorders), autoinflammatory disorders, defects in intrinsic and innate immunity, complement deficiencies, and phenocopies of inborn errors of immunity [9].

## Results

Among 4718 registered patients, 2754 patients (998 females, median age 108 months) were alive and on monthly follow-up (58.3%, before the emergence of COVID-19 first report in the country 19 February 2020). During this period, each patient had on average 8 follow-up visits in our peripheral centers, and the COVID-19 test was performed in patients with the clinical triad of cough, fever, and dyspnea. To date, 19 patients (7 females, median age 106 months) were confirmed with positive reverse transcriptase-polymerase chain reactions (RT-PCR) SARS-CoV-2 test (1:144 incidence compared to 1:178 in the total population, 1.23 folds higher risk of infections). It seems that this measurement is underestimated since PID patients/families were trained for tight isolation compared to other immunocompetent individuals in the population. Exposure to SARS-CoV-2 from an unknown source or a source outside the child’s family accounted for 84.2% of the cases of infection (15.8% of PID patients have a medical history of exposure to another COVID-19-infected family members prior to their hospital admission). Detailed comparison of infection rates between PID patients versus the total population based on adjusted age-groups is shown in Fig. S1.

Combined immunodeficiencies (*n* = 10, all without hematopoietic stem cell transplantation or HSCT, 47.0%) were the major PID entity among COVID-19 positive cases followed by humoral immunodeficiencies (*n* = 4), phagocytic defects (*n* = 2), immune dysregulation (*n* = 2), and autoinflammatory disorders (*n* = 1, Table 1). Of note, no COVID-19 infection was observed in patients with innate or complement deficiencies (117 and 85 total registered alive patients, respectively). COVID-19 infections alone or in complex with other manifestations were the first clinical presentation of PID in 4 patients, mainly combined immunodeficiencies (Tables 2 and 3). Current genetic data on the evaluated patients is indicated in Fig. S2. Of note, COVID-19 infection was not yet reported in any of the alive patients with a defect in the IFN pathway (*n* = 23, data not shown). The geographical distribution of identified patients matched with the cumulative incidence of PID, but not with the incidence of COVID-19 in the general population (Fig. S3).

**Table 1:** Epidemiologic characteristics, genetic diagnosis and outcomes of COVID-19 infection in the patients with different types of primary immunodeficiencies

Primary immunodeficiency categories	Total patients in the registry	Alive patients during the pandemic	Number of COVID-19 patients (%)	Monogenic defects of patients with COVID-19	Mortality due to COVID-19, N (%)	Mortality rate due to COVID-19
Combined immunodeficiencies	1392	630	10 (1.5)	–	6 (60.0)	0.009
Non-syndromic combined immunodeficiencies	576	247	6 (2.4)	–	5 (83.3)	0.020
Severe combined immunodeficiency	355	113	5 (4.4)	–	4 (80)	0.035
Less profound combined immunodeficiencies	221	134	1 (0.7)	STK4	1 (100)	0.007
Syndromic combined immunodeficiencies	816	383	4 (1.0)	–	1(25)	0.002
Wiskott-Aldrich syndrome	74	59	1 (1.7)	WAS	–	–
Ataxia-telangiectasia	292	86	1 (1.1)	ATM	–	–
Other syndromic combined immunodeficiencies	450	238	2 (0.8)	DNMT3B (n = 2)	1 (50)	0.004
Predominantly antibody deficiencies	1391	1002	4 (0.4)	–	–	–
Agammaglobulinemia	208	147	1 (0.6)	BTK	–	–
Common variable immunodeficiency	599	352	1 (0.2)	–	–	–
Hyper immunoglobulin M syndrome	102	86	1 (1.1)	–	–	–
Selective IgA deficiency	193	185	1 (0.1)	–	–	–
Other antibody deficiencies	285	232	0	–	–	–
Congenital defects of phagocytes	782	426	2 (0.4)	–	–	–
Chronic granulomatous disease	385	217	2 (0.9)	CYBA (n = 1)	–	–
Other phagocytosis defects	397	209	0	–	–	–
Diseases of immune dysregulation	117	90	2 (2.2)	–	1 (50)	0.011
Familial hemophagocytic lymphohistiocytosis	44	37	1 (2.7)	RAB27A	1 (100)	0.027
Susceptibility to EBV and lymphoproliferation	50	34	1 (2.9)	CD70	–	–
Other immune dysregulations	23	19	0	–	–	–
Autoinflammatory disorders	734	389	1 (0.2)	–	1 (100)	0.002
Non-inflammasome-related conditions	45	40	1	IL1RN	1 (100)	0.025
Other autoinflammatory disorders	689	549	0	–	–	–
Other primary immunodeficiencies*	302	217	0	–	–	–
Total	4718	2754	19 (0.68)	–	8 (42.1)	0.003

*Other primary immunodeficiencies include complement deficiency and innate immunodeficiencies

*EBV* Epstein-Barr virus, *STK4* serine/threonine kinase 4 gene, *WAS* WASP actin nucleation promoting factor gene, *ATM* ataxia- telangiectasia mutated gene, *BTK* Bruton’s tyrosine kinase gene, *CYBA* cytochrome B-245 alpha chain gene, *RAB27A* RAS-associated protein 27A gene, *CD70* tumor necrosis factor ligand family cluster of differentiation 70 gene, *IL1RN* interleukin 1 receptor antagonist gene

**Table 2:** Demographic data and clinical manifestation before COVID-19 infections in 19 primary immunodeficient patients

Primary immunodeficiency categories		ID	PID diagnosis	Gender	Age of onset (m)	Age of PID diagnosis (m)	Infection	Autoimmunity	Lymphoproliferation	Other PID clinical manifestation
Combined immunodeficiencies	Non-syndromic combined immunodeficiencies	P1	SCID	F	1	1	URI, LRI	–	LAP	Sensitivity to light
		P2	SCID	M	6	20	LRI	–	–	–
		P3	SCID	M	6	6	LRI	–	–	–
		P4	Omenn syndrome	M	1.5	2.5	BCGosis, LRI	–	LAP	Severe scaling erythematous skin lesions
		P5	CID	M	4	11	LRI	AIT	LAP	–
		P6	STK4	M	108	108	Meningitis, cellulitis	ITP, AIHA	LAP	Seizure, neurological disorders
	Syndromic combined immunodeficiencies	P7	WAS	M	4	4	LRI	–	–	Chronic diarrhea, microcytic thrombocytopenia
		P8	ATM	M	24	108	URI, LRI	–	–	Ataxic gait, telangiectasia
		P18	DNMT3B	M	10	17	URI, LRI	–	LAP	Recurrent diarrhea
		P19	DNMT3B	F	15	28	URI, LRI	–	–	Recurrent diarrhea, bronchiectasis
Predominantly antibody deficiencies		P9	BTK	M	36	48	Skin infection, URI, LRI	–	–	Urticaria, erythematous skin lesions
		P10	CVID	M	12	240	OME, LRI	ITP, AIHA, JIA	–	Recurrent diarrhea, bronchiectasis
		P11	HIgM	F	36	72	OME, LRI, osteomyelitis	–	HSM	FTT, granulomatous inflammatory process in BM
		P12	SIgAD	M	6	84	OME, LRI, recurrent oral herpes lesions	–	–	Bronchiectasis
Congenital defects of phagocytes		P13	CGD	F	36	36	LRI	–	–	FTT
		P14	CYBA	M	1	120	URI, LRI	–	Pulmonary granulomatosis lesion	Bronchiectasis severe pulmonary fibrosis
Diseases of immune dysregulation		P15	RAB27A	F	60	106	URI, LRI	AIHA	HSM	Albinism, gray hair, severe anal ulcer, hemophagocytic BM
		P16	CD70	F	84	108	URI, LRI	Behcet’s disease, alopecia	HSM	HL
Autoinflammatory disorders		P17	IL1RN	F	1	4	Cellulitis, dental abscess, gingivitis	UC	HSM	Rash and skin lesions, edema in the right shoulder, chronic diarrhea, severe generalized erythroderma, ascites, anemia, femur swelling

*SCID* severe combined immunodeficiency, *CID* combined immunodeficiency, *STK4* serine/threonine kinase 4 gene, *WAS* WASP actin nucleation promoting factor gene, *ATM* ataxia-telangiectasia mutated gene, *BTK* Bruton’s tyrosine kinase gene, *CYBA* cytochrome B-245 alpha chain gene, *RAB27A* RAS-associated protein 27A gene, *CD70* tumor necrosis factor ligand family cluster of differentiation 70 gene, *IL1RN* interleukin 1 receptor antagonist gene, *CVID* common variable immunodeficiency, *HIgM* hyper IgM syndrome, *SIgAD* selective immunoglobulin A deficiency, *CGD* chronic granulomatous disease, *LRI* lower respiratory infections, *URI* upper respiratory infections, *OME* otitis media with effusion, *AIT* autoimmune hypothyroidism, *JIA* juvenile idiopathic arthritis, *AIHA* autoimmune hemolytic anemia, *ITP* immune thrombocytopenic purpura, *UC* ulcerative colitis, *LAP* lymphadenopathy, *SM* splenomegaly, *HM* hepatomegaly, *HSM* hepatosplenomegaly, *FTT* failure to thrive, *BM* bone marrow aspiration/biopsy, *HL* Hodgkin’s lymphoma, *F* female, *M* male

**Table 3:** Clinical presentation after COVID-19 infections and outcome of treatment strategy in 19 primary immunodeficient patients

Primary immunodeficiency categories		ID	PID diagnosis	Age at COVID − 19 infection (m)	Clinical signs and complications after COVID-19	Organ involvement	Treatment strategy	Medications	Outcome
Combined immunodeficiencies	Non-syndromic combined immunodeficiencies	P1	SCID	10	Fever, cough, drop of oxygen saturation, respiratory distress	Lung	Hospitalized, NICU, requiring O2/NIV	Azithromycin, IVIG	Death
		P2	SCID	20	Drop of oxygen saturation	Lung	Hospitalized, NICU	Azithromycin, IVIG	Death
		P3	SCID	8	Fever, faintness, respiratory distress	Lung	Hospitalized, NICU	Hydroxychloroquine, vancomycin, meropenem, IVIG	Death
		P4	Omenn syndrome	6	Fever, tachypnea, vomiting heart failure, seizure(once), cardiomegaly, a drop of oxygen saturation, respiratory distress	Heart, Lung	Hospitalized, NICU	Isoniazid, rifampin, ethambutol, vitamin B6, cotrimoxazole	Death
		P5	CID	11	Respiratory distress, CD4 lymphopenia	Lung	Hospitalized, NICU	Hydroxychloroquine, azithromycin, IVIG	Recovery
		P6	STK4	144	Fever, loss of appetite, jaundice, abdominal pain, bloody diarrhea, cardiac and pulmonary arrest	Gastrointestinal, lung, heart	Hospitalized, requiring O2/NIV	Acyclovir, ceftriaxone, vancomycin, dexamethasone	Death
	Syndromic combined immunodeficiencies	P7	WAS	5	Fever, cough, respiratory distress	Lung	Hospitalized requiring O2/NIV	Meropenem. cotrimoxazole, vancomycin, azithromycin, IVIG	Recovery
		P8	ATM	206	Fever, diarrhea	Gastrointestinal	Hospitalized	Azithromycin, IVIG	Recovery
		P18	DNMT3B	130	Fever, cough, respiratory distress	Lung	Hospitalized requiring O2/NIV	Azithromycin, IVIG	Recovery
		P19	DNMT3B	152	Fever, dry coughs, loss of appetite, vomiting, seizure, loss of awareness, respiratory distress	Lung, gastrointestinal	Hospitalized, ICU	Azithromycin, ceftriaxone, vancomycin, IVIG	Death
Predominantly antibody deficiencies		P9	BTK	430	Dry cough, fever, sweating, abdominal pain, wheezing	Gastrointestinal, lung	Hospitalized, requiring O2/NIV	Azithromycin, IVIG	Recovery
		P10	CVID	444	Fever, dry cough, fatigue, shortness of breath, muscular pain, chest pain	Lung	Hospitalized	Hydroxychloroquine, azithromycin, meropenem, IVIG	Recovery
		P11	HIgM	72	Fever, cough	Lung	Hospitalized, requiring O2/NIV	Hydroxychloroquine, azithromycin	Recovery
		P12	SIgAD	96	Fever, mild clear rhinorrhea, mild suprasternal retraction, tachypnea, mild consolidation, productive cough, clear rhinorrhea	Lung	Hospitalized	Meropenem, clindamycin, hydroxychloroquine, IVIG	Recovery
Congenital defects of phagocytes		P13	CGD	108	Fever, cough, headache	Lung	Hospitalized, requiring O2/NIV	Cotrimoxazole, cefixime, meropenem, vancomycin	Recovery
		P14	CYBA	216	Respiratory distress, fatigue, dry cough, fever, headache, loss of sense of smell and hearing, eyelid edema, severe cardiac enlargement	Lung, olfactory and auditory, cardiovascular system	Hospitalized	Cotrimoxazole, hydroxychloroquine	Recovery
Diseases of immune dysregulation		P15	RAB27A	106	Fever, vomiting, HM, liver involvement and pitting edema, respiratory distress	Gastrointestinal	Hospitalized, ICU	Hydroxychloroquine, azithromycin	Death
		P16	CD70	372	Fatigue, dry cough, sore throat	Lung	Hospitalized, requiring O2/NIV	Hydroxychloroquine, azithromycin, IVIG	Recovery
Autoinflammatory disorders		P17	IL1RN	96	Fever, dry coughs, loss of appetite, vomiting, seizure, loss of awareness, respiratory distress	Lung, gastrointestinal	Hospitalized, ICU	Hydroxychloroquine, azithromycin	Death

*SCID* severe combined immunodeficiency, *IVIG* intravenous immunoglobulin, *m* months, *CID* combined immunodeficiency, *STK4* serine/threonine kinase 4 gene, *WAS* WASP actin nucleation promoting factor gene, *ATM* ataxia-telangiectasia mutated gene, *BTK* Bruton’s tyrosine kinase gene, *CYBA* cytochrome B-245 alpha chain gene, *RAB27A* RAS-associated protein 27A gene, *CD70* tumor necrosis factor ligand family cluster of differentiation 70 gene, *IL1RN* interleukin 1 receptor antagonist gene, *CVID* common variable immunodeficiency, *HIgM* hyper IgM syndrome, *SIgAD* selective immunoglobulin A deficiency, *CGD* chronic granulomatous disease, *NIV* non-invasive ventilation, *NICU* neonatal intensive care unit, *ICU* intensive care unit

Details of clinical manifestations and laboratory findings of patients before and after SARS-CoV-2 infections are summarized in Tables 2 and 3, S2 and S3. The majority of patients had a history of lower respiratory tract infection before COVID-19 (89.4%, except for two patients with STK4 and IL1RN deficiencies). Severe distress requiring respiratory support was documented as COVID-19 features in 10 patients from which 7 had lymphoproliferation (70%) including lymphadenopathy, hepatosplenomegaly, and non-necrotizing granulomatous inflammation. Moreover, bronchiectasis (21.0%), cardiovascular complications (10.5%), and liver failure (10.5%) were observed in patients with poor prognosis. Of note, bone marrow analysis and laboratory data of COVID-19-infected patients with immune dysregulation (P15 and P16) did not reveal hemophagocytic lymphohistiocytosis (HLH) activity at the time of the current study. Acute-phase reactant proteins were negative in 8 patients, particularly in patients with severe combined immunodeficiency (SCID) and phagocytosis defects. Imaging findings on COVID-19 pulmonary complications of PID patients varied from mild prominence of bronchovascular markings to mucus plugging, prebronchial thickening, diffuse patchy opacities, collapse/consolidations, mosaic perfusion, and ground glass interstitial disease, mirroring of the severity of diagnosed PID (Figs. S4–S15).

Among the identified PID cases with COVID-19, 8 patients deceased (42.1%, Table 1) indicating a 10-folds higher mortality rate in PID patients compared to the population (0.003 vs. 0.0003, *p* < 0.001). Of note, the most lethal COVID-19 infection among PID entities was observed in patients with SCID (0.03) and familial hemophagocytic lymphohistiocytosis (FHL, 0.027). This notion confirms the previous hypothesis that SARS-CoV-2 are more life-threatening among patient with cellular immunodeficiency and immune dysregulation with almost 150-folds higher risk of mortality. All infected patients with less profound combined immunodeficiencies, FHL, and autoinflammatory disorders succumbed to COVID-infection. Monthly intravenous immunoglobulin replacement therapy was continued during the COVID-19 infection period in 12 patients, of which 66.6% recovered from the infection (4 deceased patients had combined immunodeficiencies, Table 2).

## Discussion

The current knowledge about COVID-19 in cases with underlying immunodeficiency is scarce [18–24]. Comparing to previous data reported mainly primary antibody deficient and male patients with COVID-19 infection (Table S4), we did not observe the dominancy of infected patients in this PID category despite their high frequency among the national registry with no deceased patient. In the first observation in 7 cases from Italy, all were antibody-deficient patients with 14.2% mortality [25]. In another report from 582 children and adolescents in Europe with PCR-confirmed SARS-CoV-2 infection, 3 patients (0.5% of total cases) had a previously diagnosed PID comprising common variable immunodeficiency, congenital neutropenia, and Schimke immuno-osseous dysplasia [26].

These data resembling the findings of the recent study by Parri et al. [27] where they highlighted immunological complications may underlie 7.4–12.5% of COVID-19 patients reported from pediatric emergency departments. However, our study may strengthen the current understanding of the impact of the pandemic on all subgroups of PID and highlights the requirement of tight measurements in children affected mainly with combined immunodeficiency and immune dysregulation. Of note, several recent studies identified that lymphopenia with prominent decreased T cell (mainly CD8^+^ cytotoxic T cell) counts are associated with severe COVID-19 condition and mortality [3, 28–31]. Indeed, lymphopenia seems to correlate with the cytokine profile of the severe patients which resemble our observation in PID patients [30, 32]. On the other hand, the recent multinational cohort reported by ESID (94 patients, 37% were with mild symptoms or asymptomatic, 9.5% mortality) revealed that older patients with combined immunodeficiency (14 patients in this cohort all recovered, Table S4) manifested milder presentations compared to our patients, which may reflect different underlying diseases, but also access to optimal treatment mainly HSCT [33].

While the induction of effective cellular immunity is likely essential for the COVID-19 control, dysregulated T cell activation (by overproduction of IFN associated cytokines promoting retention of lymphocytes in lymphoid organs) may underly main immunopathology and contribute to disease severity in COVID-19 patients [32, 34–36]. Although the exposure to pathogens may be lower in PID patients due to more strict self-isolation, it should be noted that the majority of PID cases require to visit the hospital and medical centers regularly which may predispose them even more to exposure to different pathogens as well as COVID-19. Even though our approach between PID patients and the general population were similarly based on decided national COVID-19 protocols (RT-PCR test was performed in cases with a suggestive triad of fever, cough, and dyspnea), it should be declared that some infected cases from the population may be missed with this inclusion criteria. Indeed, PID patients are followed-up more regularly, whereas the infected immunocompetent individual with mild infections in the normal population may not refer to the medical centers. Therefore, future epidemiological studies in other countries are required to evaluate this notion.

Our current genetic findings suggest a higher mortality rate on special molecular defects associated with deficiency of the IL-1 receptor (DIRA deficiency), STK4 deficiency (combined immunodeficiency), and RAB27A deficiency (diseases of immune dysregulation) with COVID-19. However, this data need to be supported with future evidence from other PID cohorts worldwide since these disorders are extremely rare. The notion observed could alert physicians on whether the usage of IL-1 inhibitors, such as anakinra, might be helpful for the treatment of COVID-19, and this decision should be made individually for each patient based on their genetic diagnosis and medical condition [37]. Although admission to IL-1 inhibitor in intensive care unit admitted cases could significantly reduce the mortality rate (hazard ratio of 0.22 compared to non-receivers), this might only be indicated in patients with hemophagocytic lymphohistiocytosis, macrophage activation syndrome with certain overactivation of inflammasome pathway. Of note, recent genetic analysis of 4 young brother pairs (range, 21–32 years) with respiratory insufficiency due to severe COVID-19 suggested TLR7 deficiency with reduced production of IFN-γ, a type II IFN as the underlying cause [38]. Moreover, review literature [25, 33, 38, 39, 40, 41] on PID patients with COVID-19 infections might also indicate a possible association of the disease with mutations in *BTK* (9 patients), *IRF7* (7 patients) and *TLR4* (4 patients), genes (Table S4). Although we did not identify COVID-19 infection in any of our patients with innate immune defects, this observation may also indicate future follow-up on patients with defects in TLR and IFN pathways are required to understand the genetic predisposition and pathogenesis of COVID-19 correctly, as 23 patients with disease-causing variants and life-threatening COVID-19 have been reported recently [39].

Our findings showed that about 30% of the total Iranian population is younger than 20 years and indeed it has been evident that they presented a very low frequency of COVID-19 infections and almost no mortality compared to other age categories (Fig. S1). Whereas about 57% of PID patients are under the age of 20, and a significant number of COVID-19-infected patients were observed in this age group, and the majority of them died which indicates an obvious contrast to the population. Moreover, in the total population, 70% are over 20 years old comprising a considerable percentage of patients with SARS-CoV-2 infection and the highest COVID-19 mortality. In contrast, although about 43% of PID patients are older than 20 years (1184 patients), only three SARS-CoV-2 positive patients were recorded and none of them had died due to SARS-CoV-2 which is again markedly different from the normal population. We actually observed a 10-fold higher mortality rate and a reverse pattern of the age-structure in COVID-19-infected PID patients compared to the population. This is also indeed higher than the mortality rate documented in children and adolescents in Europe where only 4 patients died, all older than 10 years, with a crude fatality rate of 0.0069. Of note, one of those deceased patients (25%) had underlying immunodeficiency due to HSCT [26]. But it is important to highlight that recessive forms of PID might play a role in the difference observed between our PID study and the European pediatric cohort since these severe PIDs may possibly increase the incidence of more severe COVID19 cases during childhood.

## Conclusion

Although COVID-19 is generally a mild disease in children and adolescents (due to the low ACE2 receptor expression and functional adaptive immunity) [42, 43], a fraction of them including PID patients may develop severe disease requiring intensive unit care admission and even fatal outcome. Future studies may corroborate the individual risk of different PID disorders and clarify the potential need for preemptive measures for specific subsets of PID patients at high risk of a critical course of COVID19.

## Supplementary Information

ESM 1 (DOCX 27600 kb).
