# Supplementary material for: Impact of SARS-CoV-2 Pandemic on Patients with Primary Immunodeficiency
Source: J Clin Immunol. 2020 Dec 1;41(2):345–55. doi: 10.1007/s10875-020-00928-x (PMC7707812; doi:10.1007/s10875-020-00928-x)
Supplement: Supplementary file 1 — (DOCX 27600 kb). [file 10875_2020_928_MOESM1_ESM.docx]

**Supplementary data**

**Impact of SARS CoV-2 Pandemic on Patients with Primary Immunodeficiency**

| **Table S1**- The 269 genes included in the panel for targeted next generation sequencing. | | | | | |
| --- | --- | --- | --- | --- | --- |
| **Genes symbols** | | | | | |
| *ACP5* | *CD81* | *GFI1* | *MAP3K14* | *RNASEH2C* | *TRAF3IP2* |
| *ACTB* | *CD8A* | *GUCY2C* | *MASP1* | *RNF168* | *TREX1* |
| *ADA* | *CEBPE* | *HAX1* | *MASP2* | *RORC* | *TRNT1* |
| *ADAM17* | *CFB* | *HPS1* | *MCM4* | *RNF31* | *TPP1* |
| *ADAR* | *CFD* | *HPS4* | *MEFV* | *RPSA* | *TPP2* |
| *AICDA* | *CFH* | *HPS6* | *MRE11A* | *RTEL1* | *TTC37* |
| *AIRE* | *CFHR1* | *ICOS* | *MSH6* | *SAMHD1* | *TTC7A* |
| *AK2* | *CFHR2* | *IFIH1* | *MS4A1* | *SBDS* | *TYK2* |
| *AP3B1* | *CFHR3* | *IFNG* | *MTHFD1* | *SEMA3E* | *UNC119* |
| *APOL1* | *CFHR4* | *IFNGR1* | *MVK* | *SERPING1* | *UNC13D* |
| *ATM* | *CFHR5* | *IFNGR2* | *MYD88* | *SH2D1A* | *UNC93B1* |
| *B2M* | *CFI* | *IGLL1* | *NBN* | *SH3BP2* | *UNG* |
| *BLM* | *CFP* | *IKBKB* | *NCF1* | *SKIV2L* | *USB1* |
| *BLNK* | *CHD7* | *IKBKG* | *NCF2* | *SCL29A3* | *VPS13B* |
| *BTK* | *CIITA* | *IKZF1* | *NCF4* | *SLC35C1* | *VPS45* |
| *C1R* | *CLPB* | *IL10* | *NFAT5* | *SLC37A4* | *WIPF1* |
| *C1QA* | *CLEC7A* | *IL10RA* | *NFKBIA* | *SLC46A1* | *XIAP* |
| *C1QB* | *COLEC11* | *IL10RB* | *NFKB2* | *SMARCAL1* | *ZAP70* |
| *C1QC* | *CORO1A* | *IL12B* | *NHP2* | *SP110* | *ZBTB24* |
| *C1S* | *CR2* | *IL12RB1* | *NLRC4* | *STAT1* |  |
| *C2* | *CSF2RA* | *IL17F* | *NLRP3* | *STAT2* |  |
| *C3* | *CSF3R* | *IL17RA* | *NLRP12* | *STAT3* |  |
| *C4A* | *COH1* | *IL17RC* | *NOD2* | *STAT5B* |  |
| *C4B* | *COPA* | *IL1RN* | *NOD10* | *STIM1* |  |
| *C5* | *CORO1A* | *IL21* | *NRAS* | *STK4* |  |
| *C6* | *CTLA4* | *IL21R* | *ORAI1* | *STX11* |  |
| *C7* | *CTPS1* | *IL2RA* | *PARN* | *SPINK5* |  |
| *C8A* | *CTSC* | *IL2RG* | *PIK3CD* | *STXBP2* |  |
| *C8B* | *CXCR4* | *IL36RN* | *PIK3R1* | *TAP1* |  |
| *C8G* | *CYBA* | *IL6* | *PLCG2* | *TAP2* |  |
| *C9* | *CYBB* | *IL7R* | *PMS2* | *TAPBP* |  |
| *CARD9* | *DCLRE1B* | *INO80* | *PNP* | *TAZ* |  |
| *CARD11* | *DCLRE1C* | *IRAK4* | *POLE* | *TBK1* |  |
| *CARD14* | *DKC1* | *IRF7* | *PRF1* | *TBX1* |  |
| *CASP10* | *DNMT3B* | *IRF8* | *PRKCD* | *TCF3* |  |
| *CASP8* | *DOCK8* | *ISG15* | *PRKDC* | *TCN2* |  |
| *CCBE1* | *ELANE* | *ITCH* | *PSMB8* | *TERT* |  |
| *CD17* | *EPG5* | *ITGB2* | *PSTPIP1* | *THBD* |  |
| *CD19* | *FADD* | *ITK* | *PTPRC* | *TICAM1* |  |
| *CD27* | *FAS* | *JAGN1* | *RAB27A* | *TINF2* |  |
| *CD247* | *FASLG* | *JAK3* | *RAC2* | *TLR3* |  |
| *CD3D* | *FCGR3A* | *KRAS* | *RAG1* | *TMC6* |  |
| *CD3E* | *FCN3* | *LAMTOR2* | *RAG2* | *TMC8* |  |
| *CD3G* | *FERMT1* | *LCK* | *RBCK1* | *TMEM173* |  |
| *CD40* | *FERMT3* | *LIG4* | *RFX5* | *TNFRSF13B* |  |
| *CD40LG* | *FOXN1* | *LPIN2* | *RFXANK* | *TNFRSF13C* |  |
| *CD46* | *FOXP3* | *LRBA* | *RFXAP* | *TNFRSF1A* |  |
| *CD59* | *FPR1* | *LYST* | *RHOH* | *TNFRSF4* |  |
| *CD79A* | *G6PC3* | *MAGT1* | *RNASEH2A* | *TNFSF12* |  |
| *CD79B* | *GATA2* | *MALT1* | *RNASEH2B* | *TRAF3* |  |

**Table S2-** General laboratory tests during COVID-19 infections in 17 primary immunodeficient patients.

| **ID** | **PID diagnosis** | **Age (m)** | **WBC (cells/ul)** | **Lymph-count (cells/ul)** | **Lymph**  **(%)** | **PMN-count (cells/ul)** | **PMN**  **(%)** | **Hb (g/dL)** | **PLT (× 10^9^/L)** | **CRP (mg/dL)** | **ESR (mm/hr)** |
| --- | --- | --- | --- | --- | --- | --- | --- | --- | --- | --- | --- |
| P1 | SCID | 10 | 4900 | 1470 | 30 | 3136 | 64 | 7.5 | 82 | Negative | 55 |
| P2 | SCID | 20 | 1900 | 570 | 30 | 1064 | 56 | 7.9 | 55 | Negative | 43 |
| P3 | SCID | 8 | 1210 | 145 | 12 | 992.2 | 82 | 8.3 | 56 | Negative | 50 |
| P4 | Omenn syndrome | 6 | 1000 | 160 | 16 | 340 | 34 | 12.8 | 618 | 44.5 | 59 |
| P5 | CID | 11 | 15370 | 4395 | 28 | 8976 | 58 | 12.9 | 381 | 40 | 63 |
| P6 | STK4 | 144 | 13100 | 1703 | 13 | 6419 | 49 | 6.4 | 414 | 48 | >120 |
| P7 | WAS | 5 | 19600 | 6624 | 33 | 10956 | 56 | 9.1 | 88 | 32.1 | 46 |
| P8 | ATM | 206 | 8800 | 1408 | 16 | 6160 | 70 | 15.4 | 302 | 3 | 12 |
| P18 | DNMT3B | 130 | 5950 | 2890 | 48 | 2440 | 41 | 11.8 | 209 | 45 | 64 |
| P19 | DNMT3B | 152 | 3700 | 1170 | 32 | 2265 | 62 | 10.5 | 180 | 60 | 79 |
| P9 | BTK | 430 | 7370 | 2351 | 31 | 4156 | 56 | 12.6 | 285 | Negative | 4 |
| P10 | CVID | 444 | 11000 | 4620 | 42 | 5390 | 49 | 14.5 | 146 | 45 | 75 |
| P11 | HIgM | 72 | 5800 | 1276 | 22 | 4060 | 70 | 7.9 | 74 | 52 | 140 |
| P12 | SIgAD | 96 | 10300 | 3399 | 33 | 3687 | 35 | 10.2 | 679 | 85 | 135 |
| P13 | CGD | 108 | 12200 | 3904 | 32 | 7320 | 60 | 10.2 | 627 | 1 | 86 |
| P14 | CYBA | 216 | 6500 | 2665 | 41 | 3250 | 50 | 11.5 | 320 | 3 | 56 |
| P15 | RAB27A | 106 | 6500 | 5323 | 82 | <10 | <1 | 7.9 | 15 | Negative | 5 |
| P16 | CD70 | 372 | 5400 | 1404 | 26 | 3564 | 66 | 12.2 | 232 | 61 | 113 |
| P17 | IL1RN | 96 | 28300 | 13867 | 49 | 7782 | 27 | 9.5 | 262 | 76 | 142 |

*SCID: Severe combined immunodeficiency, m: months, CID: combined immunodeficiency, STK4: Serine/threonine kinase 4 gene, WAS: WASP actin nucleation promoting factor gene, ATM: Ataxia-* *telangiectasia mutated gene, BTK: Bruton's tyrosine kinase gene, CYBA: Cytochrome B-245 alpha chain gene, RAB27A: RAS-associated protein 27A gene, CD70: tumor necrosis factor ligand family cluster of differentiation 70 gene, IL1RN: Interleukin 1 receptor antagonist gene, CVID:* *Common variable immunodeficiency, HIgM: Hyper IgM syndrome, SIgAD: Selective immunoglobulin A deficiency, CGD: Chronic granulomatous disease, WBC: white blood cells, Lymph: lymphocytes, PMN: polymorphonuclear leukocytes, Hb:* *hemoglobin, PLT: platelets, CRP: C-reactive protein, ESR: Erythrocyte sedimentation rate.*

**Table S3-** Immunologic investigation of 17 primary immunodeficient patients infected by COVID-19.

| **ID** | **PID diagnosis** | **Lymph-count (cell/ul)** | **CD3-count** | **CD3 (%)** | **CD4-count** | **CD4 (%)** | **CD8-count** | **CD8 (%)** | **CD16-count** | **CD16 (%)** | **CD19-count** | **CD19 (%)** | **IgG (mg/dl)** | **IgA (mg/dl)** | **IgM (mg/dl)** | **IgE (IU/dl)** |
| --- | --- | --- | --- | --- | --- | --- | --- | --- | --- | --- | --- | --- | --- | --- | --- | --- |
| P1 | SCID T- B- NK+ | 1470 | 882 | 60 | 514 | 35 | 264 | 18 | 294 | 20 | 44 | 3 | 109 | 60 | 116 | 512 |
| P2 | SCID T- B+ NK+ | 570 | 74 | 13 | 51 | 9.0 | 10 | 1.8 | 256 | 45 | 239 | 42 | 1341 | 320 | 204 | 152 |
| P3 | SCID T- B+NK- | 145 | 59 | 41 | 30 | 21 | 29 | 20 | 13 | 9 | 69 | 48 | 184 | 29 | 70 | 15 |
| P4 | Omenn syndrome | 160 | 90 | 56 | 55 | 34 | 37 | 23 | 54 | 34 | 3 | 2.3 | 711 | 41 | 12 | 311 |
| P5 | CID | 4395 | 345 | 7.8 | 167 | 3.8 | 149 | 3.4 | 435 | 9.9 | 3196 | 72 | 342 | 24 | 15 | 5 |
| P6 | STK4 | 1703 | 1560 | 89 | 306 | 18 | 1158 | 68 | 85 | 5 | 34 | 2 | 2125 | 1260 | 116 | 277 |
| P7 | WAS | 6624 | NI | NI | NI | NI | NI | NI | NI | NI | NI | NI | 1298 | 92 | 29 | 128 |
| P8 | ATM | 1408 | 675 | 48 | 337 | 24 | 253 | 18 | 490 | 34 | 77 | 5 | 766 | 13 | 85 | 10 |
| P18 | DNMT3B | 2890 | 1880 | 65 | 1205 | 42 | 610 | 21 | 720 | 25 | 170 | 6 | 229 | <10 | 24 | 1 |
| P19 | DNMT3B | 1170 | 830 | 71 | 468 | 40 | 327 | 28 | 235 | 20 | 59 | 4.5 | 190 | <7 | <10 | 0 |
| P9 | BTK | 2351 | 2139 | 91 | 1199 | 51 | 705 | 30 | 175 | 7 | 23 | <2 | 264 | <10 | 75 | 3.7 |
| P10 | CVID | 4620 | 3010 | 65 | 1709 | 37 | 1247 | 27 | 605 | 13 | 368 | 8 | 145 | <40 | <60 | 5 |
| P11 | HIgM | 1276 | 931 | 73 | 535 | 42 | 382 | 30 | 191 | 15 | 127 | 10 | 862 | 68.5 | 3715 | 1 |
| P12 | SIgAD | 3399 | 2277 | 67 | 689 | 20 | 1610 | 47 | 407 | 12 | 679 | 20 | 1622 | 2 | 38 | 1 |
| P13 | CGD | 3904 | NI | NI | NI | NI | NI | NI | NI | NI | NI | NI | 1990 | <10 | 172 | 513 |
| P14 | CYBA | 2665 | 1937 | 72 | 1125 | 43 | 799 | 30 | 133 | 5 | 559 | 21 | 1211 | 135 | 90 | 34 |
| P15 | RAB27A | 5323 | NI | NI | NI | NI | NI | NI | NI | NI | NI | NI | NI | NI | NI | NI |
| P16 | CD70 | 1404 | 1010 | 72 | 421 | 30 | 463 | 33 | 126 | 8.9 | 151 | 15 | 191 | 72 | 37 | 5 |
| P17 | IL1RN | 13867 | 10122 | 73 | 5408 | 39 | 3189 | 23 | 1941 | 14 | 1664 | 12 | 1118 | 50 | 69 | 59 |

*SCID: Severe combined immunodeficiency, m: months, CID: combined immunodeficiency, STK4: Serine/threonine kinase 4 gene, WAS: WASP actin nucleation promoting factor gene, ATM: Ataxia-* *telangiectasia mutated gene, BTK: Bruton's tyrosine kinase gene, CYBA: Cytochrome B-245 alpha chain gene, RAB27A: RAS-associated protein 27A gene, CD70: tumor necrosis factor ligand family cluster of differentiation 70 gene, IL1RN: Interleukin 1 receptor antagonist gene, CVID:* *Common variable immunodeficiency, HIgM: Hyper IgM syndrome, SIgAD: Selective immunoglobulin A deficiency, CGD: Chronic granulomatous disease, Lymph: lymphocytes, NI: Not indicated.*

**Table S4-** Currently primary immunodeficient patients reported by COVID-19.

| **PID diagnosis** | **Gender/Age** | **Consequence** | **Reference** |
| --- | --- | --- | --- |
| Agammaglobulinemia | Male/ 56 y | Recovery | Quinti et al [26] |
| BTK deficiency | Male/ 34y | Recovery | Quinti et al [26] |
| CVID | Female/ 59y | ICU admission, death | Quinti et al [26] |
| CVID | Female/ 32y | Recovery | Quinti et al [26] |
| CVID | Male/ 57y | ICU admission, recovery | Quinti et al [26] |
| CVID | Male/ 52y | Recovery | Quinti et al [26] |
| CVID | Male/ 41y | ICU admission, recovery | Quinti et al [26] |
| CVID | NR | Recovery | Gotzinger et al [27] |
| Congenital neutropenia | NR | Recovery | Gotzinger et al [27] |
| Schimke immuno-osseous dysplasia | NR | Recovery | Gotzinger et al [27] |
| Hypogammaglobulinemia | Male/7 y | Cardiomyopathy, and chronic pulmonary disease | Dinkelbach et al [21] |
| BTK deficiency | Male/34y | Interstitial pneumonia, recovery | Soresina et al [22] |
| BTK deficiency | Male/26y | Interstitial pneumonia, recovery | Soresina et al [22] |
| TLR7 deficiency | Male/32y | ICU admission, recovery | van der Made et al [39] |
| TLR7 deficiency | Male/29y | ICU admission, death | van der Made et al [39] |
| TLR7 deficiency | Male/21y | ICU admission, recovery | van der Made et al [39] |
| TLR7 deficiency | Male/23y | ICU admission, recovery | van der Made et al [39] |
| MAS/HLH | Female/8y | Organomegaly, recovery | Klocperk et al [43] |
| ARPC1B deficiency | Male/8 m | Septic shock, recovery | Castano-Jaramillo et al [44] |
| Antibody deficiency with syndromic features | Male/40y | Pneumothorax, pulmonary hypertension, ICU admission, death | Meyts et al [34] |
| CVID | Female/40y | Renal failure, death | Meyts et al [34] |
| CVID | Female/60y | Renal failure, ICU admission, death | Meyts et al [34] |
| CVID | Female/60y | Sepsis, ICU admission, death | Meyts et al [34] |
| IgG subclass deficiency | Female/>70y | Renal failure, ICU admission, death | Meyts et al [34] |
| IgG subclass and IgA deficiency | Male/>70y | Renal failure, ICU admission, death | Meyts et al [34] |
| CVID | Female/>70y | Renal failure, death | Meyts et al [34] |
| CYBB deficiency | Male/2y | HLH, ICU admission, death | Meyts et al [34] |
| XIAP deficiency | Male/15y | HLH, sepsis, ICU admission, death | Meyts et al [34] |
| NFKB2 deficiency | Male/40y | ICU admission, recovery | Meyts et al [34] |
| Agammaglobulinemia | Male/40y | ICU admission, recovery | Meyts et al [34] |
| CVID | Male/50y | ICU admission, recovery | Meyts et al [34] |
| NFKB2 deficiency | Male/15y | ICU admission, recovery | Meyts et al [34] |
| CVID | Male/40y | ICU admission, recovery | Meyts et al [34] |
| CID Trisomy 21 | Male/10y | ICU admission, recovery | Meyts et al [34] |
| Wiskott- Aldrich syndrome | Male/10y | ICU admission, recovery | Meyts et al [34] |
| CID Trisomy 21 | Female/2y | ICU admission, recovery | Meyts et al [34] |
| BTK deficiency | Male/10y | Recovery | Meyts et al [34] |
| CVID | Female/30y | Recovery | Meyts et al [34] |
| CVID | Male/30y | Recovery | Meyts et al [34] |
| CVID | Male/40y | Recovery | Meyts et al [34] |
| CVID | Male/40y | Recovery | Meyts et al [34] |
| Hypogammaglobulinemia | Female/40y | Recovery | Meyts et al [34] |
| CVID | Male/40y | Recovery | Meyts et al [34] |
| ARPC1B deficiency | Male/2y | Recovery | Meyts et al [34] |
| CID Trisomy 21 | Male/10y | Recovery | Meyts et al [34] |
| DiGeorge syndrome | Male/2y | Recovery | Meyts et al [34] |
| MEFV deficiency | Male/60y | Recovery | Meyts et al [34] |
| Immune dysregulation and autoinflammation | Male/40y | Recovery | Meyts et al [34] |
| PEPD deficiency | Male/30y | Recovery | Meyts et al [34] |
| CTLA4 deficiency | Female/15y | Recovery | Meyts et al [34] |
| CTLA4 deficiency | NR/30y | Recovery | Meyts et al [34] |
| CVID | Male/30y | Recovery | Meyts et al [34] |
| IgG subclass deficiency | Female/60y | Recovery | Meyts et al [34] |
| Wiskott- Aldrich syndrome | Male/2y | Asymptomatic-mild | Meyts et al [34] |
| Immune dysregulation ALPS like | Male/15y | Recovery | Meyts et al [34] |
| CMCC | Male/2y | Recovery | Meyts et al [34] |
| IFNGR2 deficiency | Male/2y | Recovery | Meyts et al [34] |
| DNAJC2 deficiency | Male/10y | Recovery | Meyts et al [34] |
| Hypogammaglobulinemia | Male/10y | Asymptomatic-mild | Meyts et al [34] |
| Antibody deficiency with syndromic features | Male/10y | Recovery | Meyts et al [34] |
| CVID | Male/15y | Recovery | Meyts et al [34] |
| IL2RG deficiency | Male/20y | Asymptomatic-mild | Meyts et al [34] |
| BTK deficiency | Male/20y | Recovery | Meyts et al [34] |
| CVID | Male/30y | Asymptomatic-mild | Meyts et al [34] |
| CVID | Male/30y | Asymptomatic-mild | Meyts et al [34] |
| CVID | Female/30y | Recovery | Meyts et al [34] |
| CVID | Male/30y | Asymptomatic-mild | Meyts et al [34] |
| CVID | Male/30y | Asymptomatic-mild | Meyts et al [34] |
| BTK deficiency | Male/30y | Asymptomatic-mild | Meyts et al [34] |
| PIK3R1 deficiency | Female/30y | Asymptomatic-mild | Meyts et al [34] |
| CVID | Female/40y | Asymptomatic-mild | Meyts et al [34] |
| NFKB1 deficiency | Male/40y | Recovery | Meyts et al [34] |
| BTK deficiency | Male/40y | Recovery | Meyts et al [34] |
| CVID | Female/40y | Asymptomatic-mild | Meyts et al [34] |
| CVID | Female/40y | Recovery | Meyts et al [34] |
| BTK deficiency | Male/50y | Asymptomatic-mild | Meyts et al [34] |
| BTK deficiency | Male/50y | Recovery | Meyts et al [34] |
| CVID | Male/50y | Asymptomatic-mild | Meyts et al [34] |
| NFKB1 deficiency | Female/60y | Recovery | Meyts et al [34] |
| CVID | Male/60y | Asymptomatic-mild | Meyts et al [34] |
| CVID | Male/60y | Recovery | Meyts et al [34] |
| CVID | Female/60y | Asymptomatic-mild | Meyts et al [34] |
| Agammaglobulinemia | Male/60y | Asymptomatic-mild | Meyts et al [34] |
| Hypogammaglobulinemia | Female/70y | Asymptomatic-mild | Meyts et al [34] |
| CVID | Female/70y | Asymptomatic-mild | Meyts et al [34] |
| CVID | Female/70y | Asymptomatic-mild | Meyts et al [34] |
| CVID | Female/70y | Recovery | Meyts et al [34] |
| CVID | Female/70y | Asymptomatic-mild | Meyts et al [34] |
| IgG subclass deficiency | Female/>75y | Asymptomatic-mild | Meyts et al [34] |
| Hypogammaglobulinemia | Female/>75y | Recovery | Meyts et al [34] |
| CID | Female/10y | Recovery | Meyts et al [34] |
| ZAP70 deficiency | Female/15y | Recovery | Meyts et al [34] |
| CID | Female/15y | Asymptomatic-mild | Meyts et al [34] |
| CID | Female/30y | Asymptomatic-mild | Meyts et al [34] |
| PGM3 deficiency | Male/10y | Asymptomatic-mild | Meyts et al [34] |
| STAT3 deficiency | Male/30y | Asymptomatic-mild | Meyts et al [34] |
| STAT3 deficiency | Male/40y | Asymptomatic-mild | Meyts et al [34] |
| MEFV deficiency | Female/40y | Asymptomatic-mild | Meyts et al [34] |
| MEFV deficiency | Female/50y | Asymptomatic-mild | Meyts et al [34] |
| RNASEH2B deficiency | Male/10y | Asymptomatic-mild | Meyts et al [34] |
| RNASEH2B deficiency | Male/10y | Asymptomatic-mild | Meyts et al [34] |
| SAMHD1 deficiency | Female/10y | Asymptomatic-mild | Meyts et al [34] |
| PRKCD deficiency | Male/10y | Recovery | Meyts et al [34] |
| Somatic ALPS | Female/10y | Asymptomatic-mild | Meyts et al [34] |
| LRBA deficiency | Male/20y | Recovery | Meyts et al [34] |
| AIRE deficiency | Male/20y | Recovery | Meyts et al [34] |
| CYBB deficiency | Male/10y | Recovery | Meyts et al [34] |
| NCF2 deficiency | Female/10y | Asymptomatic-mild | Meyts et al [34] |
| Phagocyte defects | Male/30y | Asymptomatic-mild | Meyts et al [34] |
| Phagocyte defects | Male/40y | Asymptomatic-mild | Meyts et al [34] |
| CYBB deficiency | Male/50y | Asymptomatic-mild | Meyts et al [34] |
| STAT1 gain-of-function | Female/10y | Asymptomatic-mild | Meyts et al [34] |
| GATA2 deficiency | Female/15y | Recovery | Meyts et al [34] |
| TLR3 deficiency | Male/40y | Recovery | Zhang et al [40] |
| TLR3 deficiency | Male/68y | Recovery | Zhang et al [40] |
| TLR3 deficiency | Male/77y | Recovery | Zhang et al [40] |
| TLR3 deficiency | Male/56y | Recovery | Zhang et al [40] |
| UNC93B1 deficiency | Male/48y | Recovery | Zhang et al [40] |
| TICAM1 deficiency | Male/49y | Recovery | Zhang et al [40] |
| TICAM1 deficiency | Female/61y | Recovery | Zhang et al [40] |
| TICAM1 deficiency | Female/71y | Death | Zhang et al [40] |
| TBK1 deficiency | Female/46y | Recovery | Zhang et al [40] |
| TBK1 deficiency | Male/17y | Recovery | Zhang et al [40] |
| IRF3 deficiency | Female/23y | Recovery | Zhang et al [40] |
| IRF3 deficiency | Female/60y | Recovery | Zhang et al [40] |
| IRF7 deficiency | Female/49y | Recovery | Zhang et al [40] |
| IRF7 deficiency | Male/50y | Recovery | Zhang et al [40] |
| IRF7 deficiency | Male/60y | Recovery | Zhang et al [40] |
| IRF7 deficiency | Male/44y | Recovery | Zhang et al [40] |
| IRF7 deficiency | Male/41y | Recovery | Zhang et al [40] |
| IRF7 deficiency | Male/69y | Recovery | Zhang et al [40] |
| IRF7 deficiency | Male/37y | Recovery | Zhang et al [40] |
| IFNAR1 deficiency | Male/38y | Recovery | Zhang et al [40] |
| IFNAR1 deficiency | Male/26y | Death | Zhang et al [40] |
| IFNAR1 deficiency | Female/23y | Recovery | Zhang et al [40] |
| IFNAR2 deficiency | Female/54y | Recovery | Zhang et al [40] |

*CVID: Common variable immunodeficiency, XLA: X-lined Agammaglobulinemia, MAS/HLH: Macrophage activation syndrome/secondary hemophagocytic lymphohistiocytosis, NR: Not reported, MSMD: Mendelian susceptibility to mycobacterial disease, ALPS: autoimmune lymphoproliferative. CGD: chronic granulomatous disease, ICU: intensive care unit. CMC: chronic mucocutaneous candidiasis,*

**Figure S1-** Comparison of COVID-19 infection and mortality rates between PID patients versus total population based on adjusted age-groups.

**
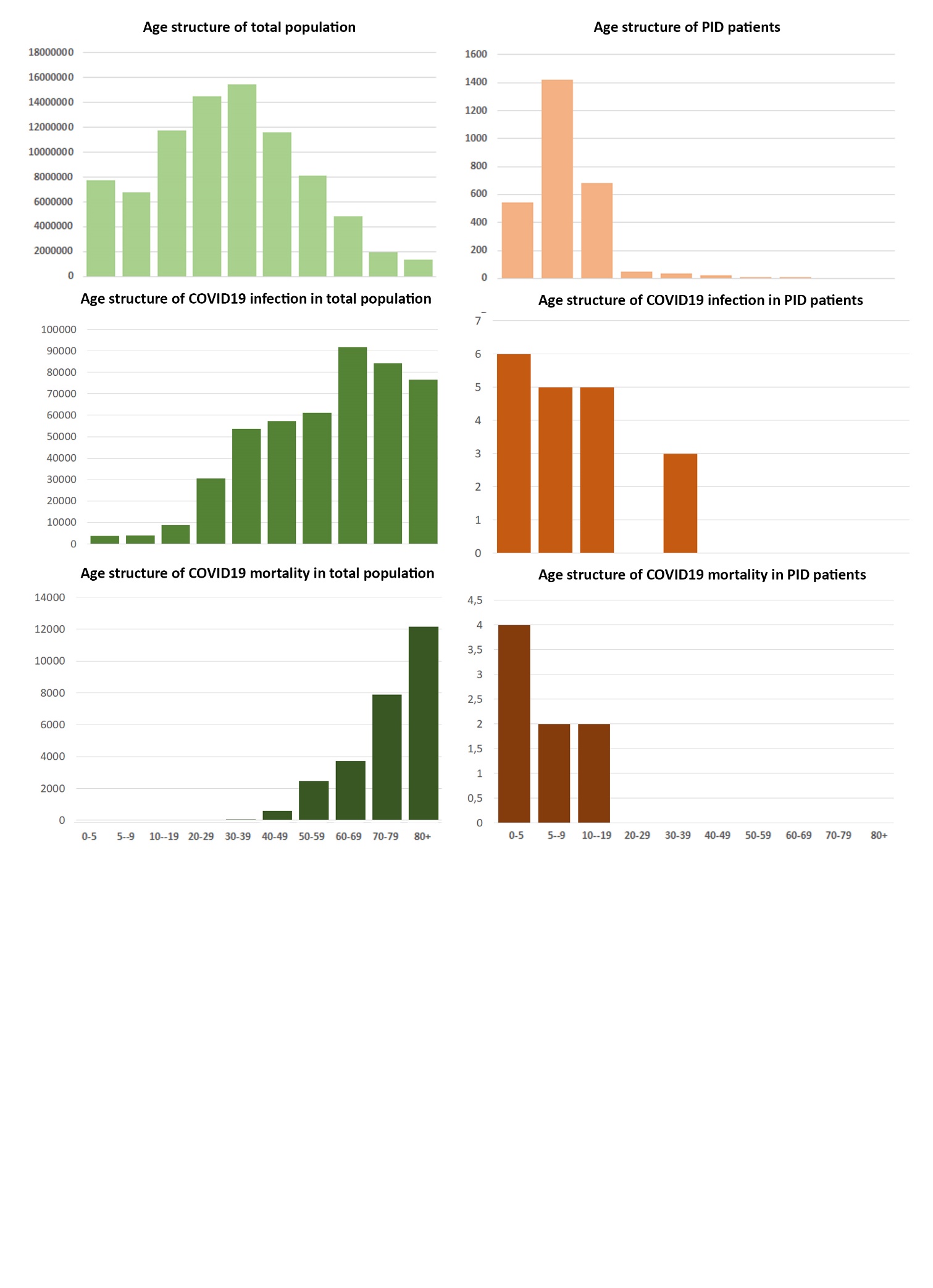
**

**Figure S2-** Percentage of COVID-19 infection among registered monogenic patients in 6 different categories of primary immunodeficiencies. Number depicted on the top of each bars represent the total number of patients with that specific gene mutation in the national PID registry with or without COVID-19 infection.

**
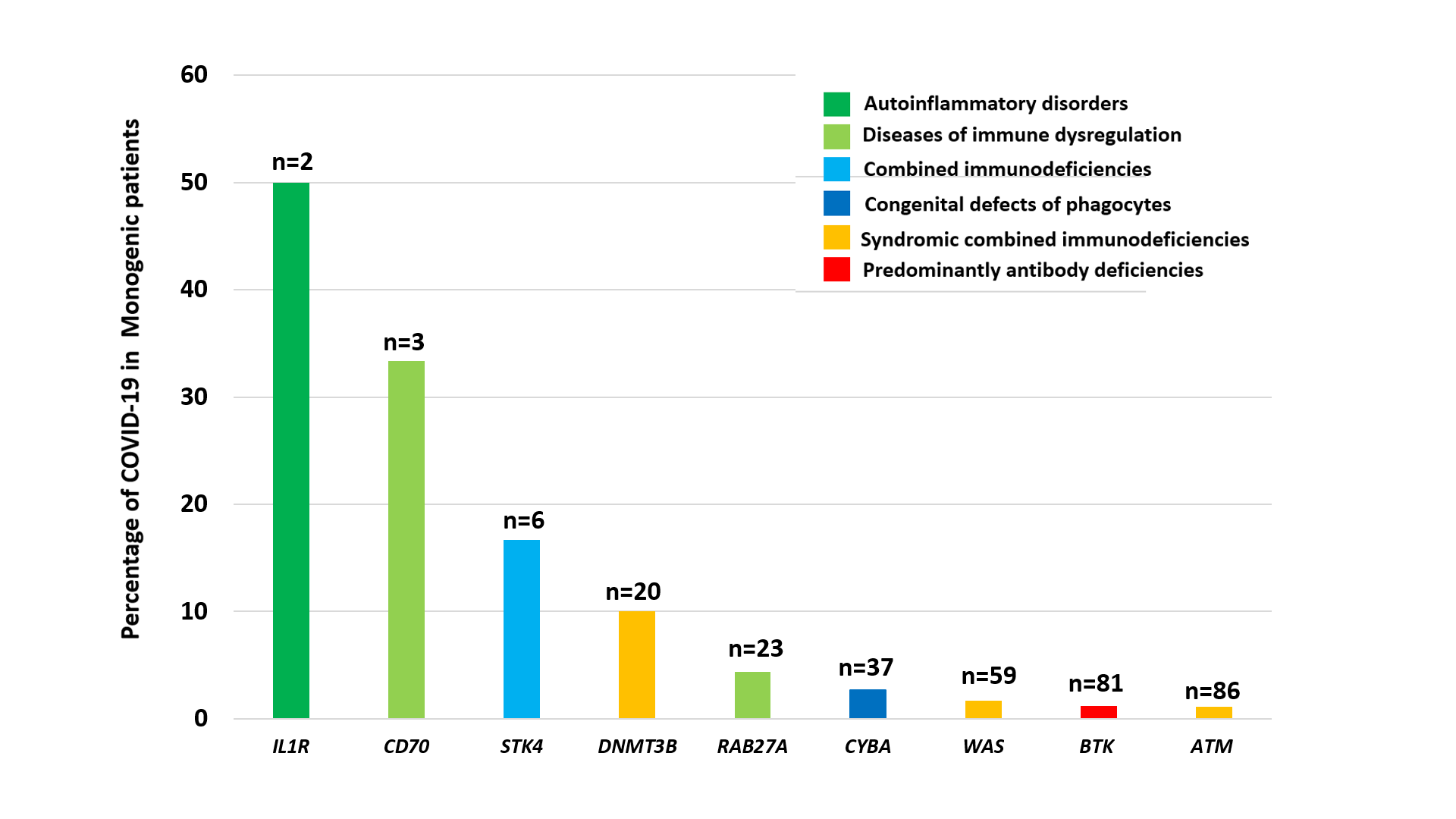
**

**Figure S3-** Schematic map of Iran indicating the cumulative incidence of PID (per million), incidence of COVID-19 (per million) and PID-COVID-19 patients in each province.

**
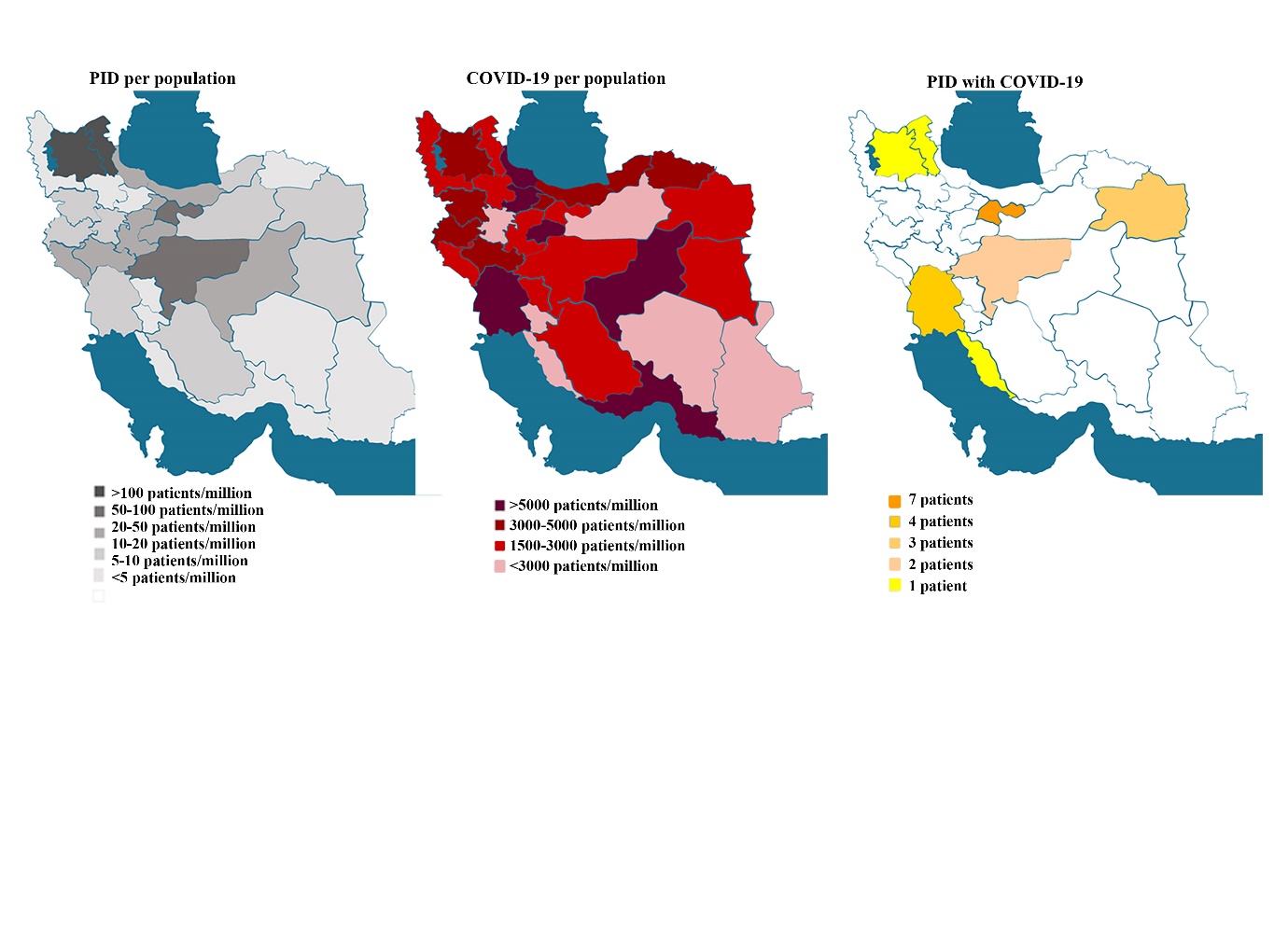
**

**Figure S4-** Chest X-ray of P2, 20 months- male with SCID and COVID-19 infections leading to death.

**
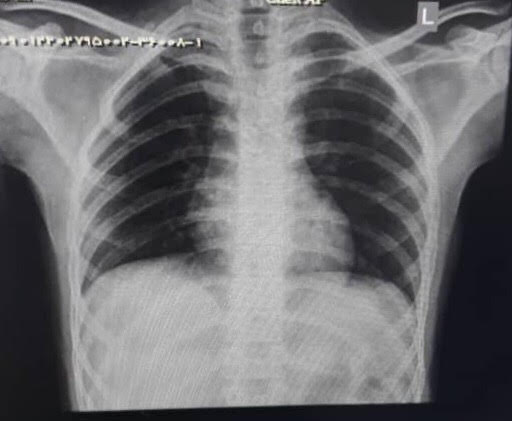
**

**Figure S5-** Chest computed tomography of P3, 8 months- male with SCID and COVID-19 infections leading to death.

**
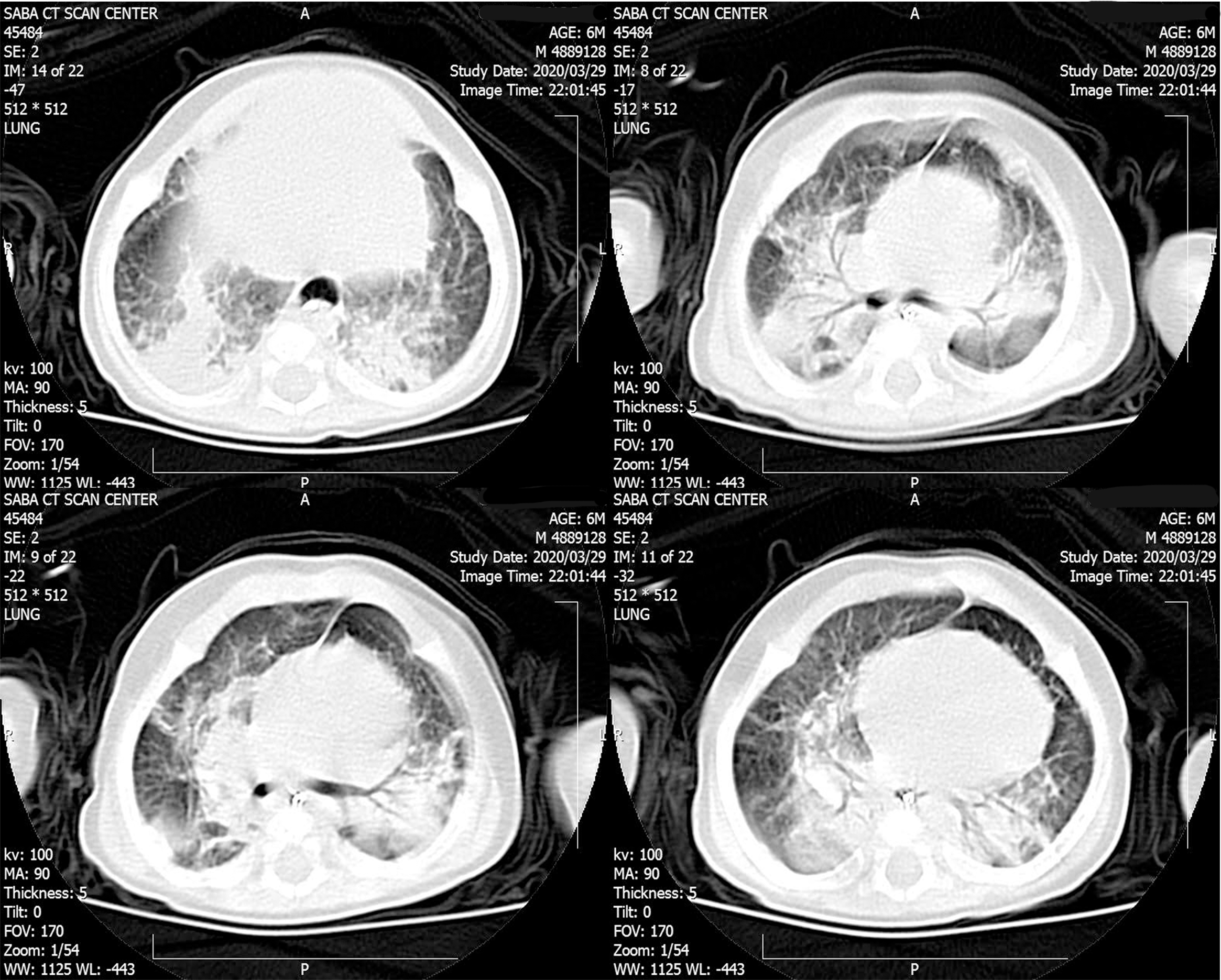
**

**Figure S6-** Chest X-ray of P4, 6 months- male with Omenn syndrome and COVID-19 infections leading to death.


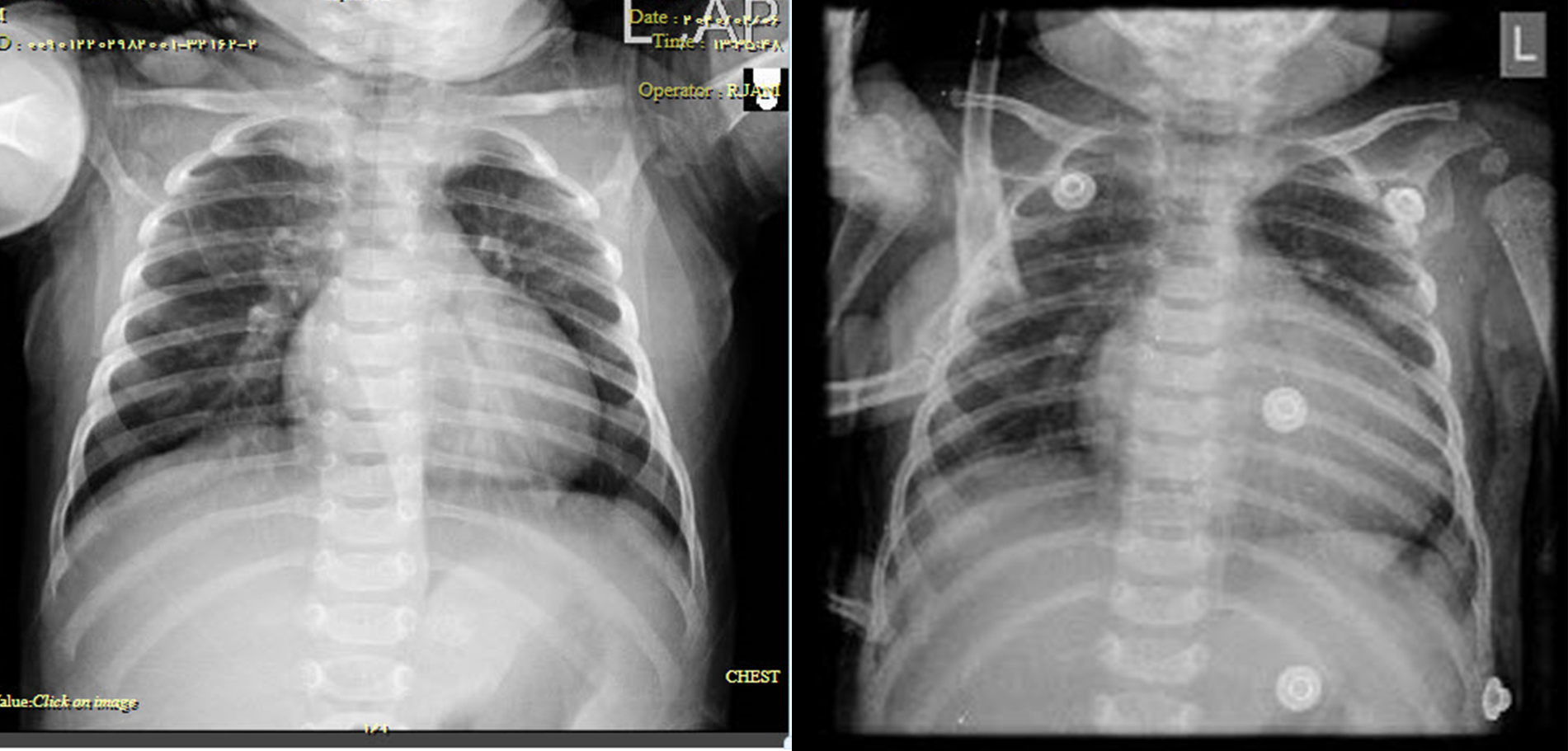


**Figure S7-** Chest computed of P5, 11 months- male with CID and COVID-19 infections leading to the recovery.

**
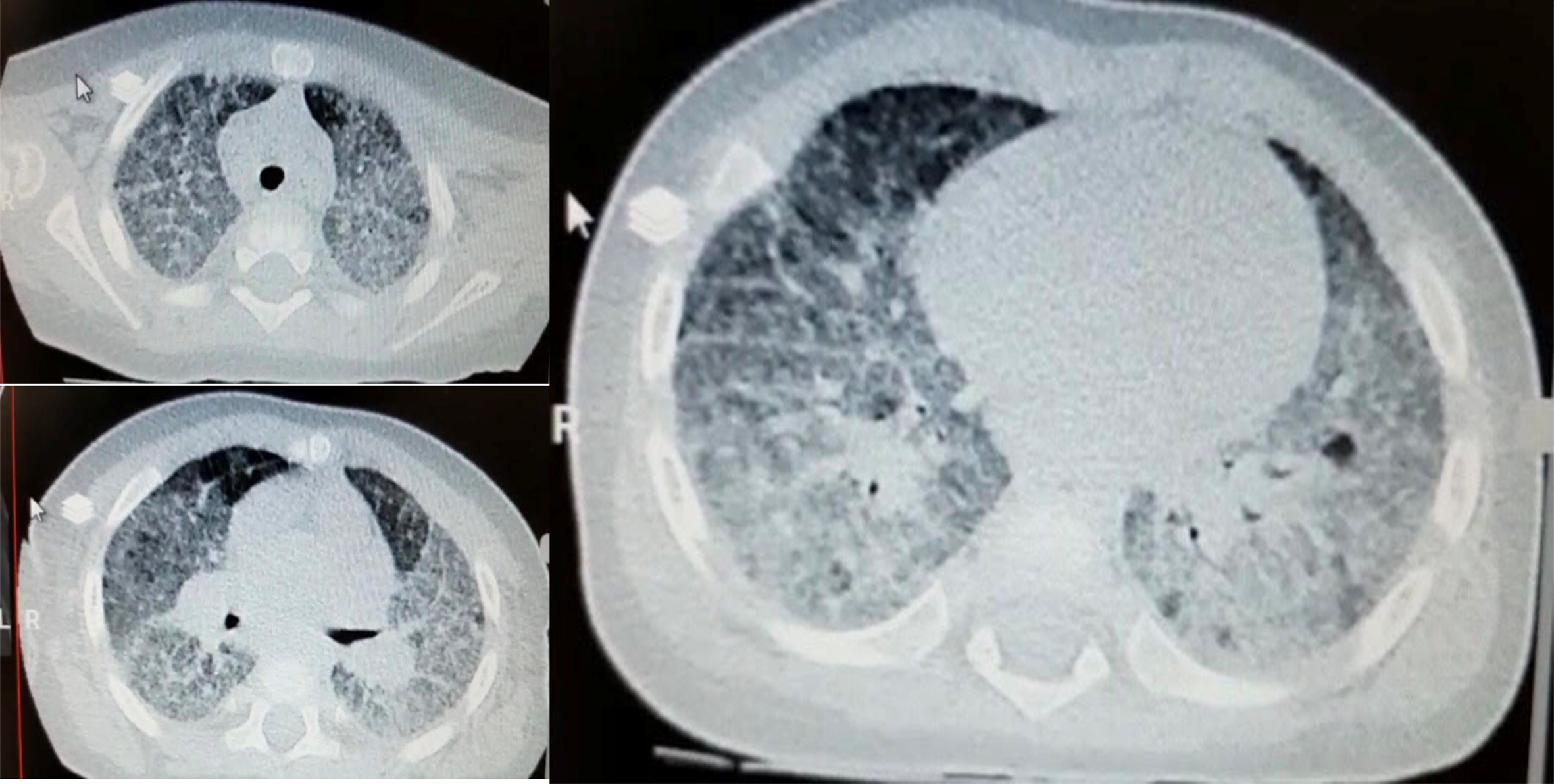
**

**Figure S8-** Chest Xray of P7, 5 months- male with Wiskott–Aldrich syndrome and COVID-19 infections leading to the recovery.


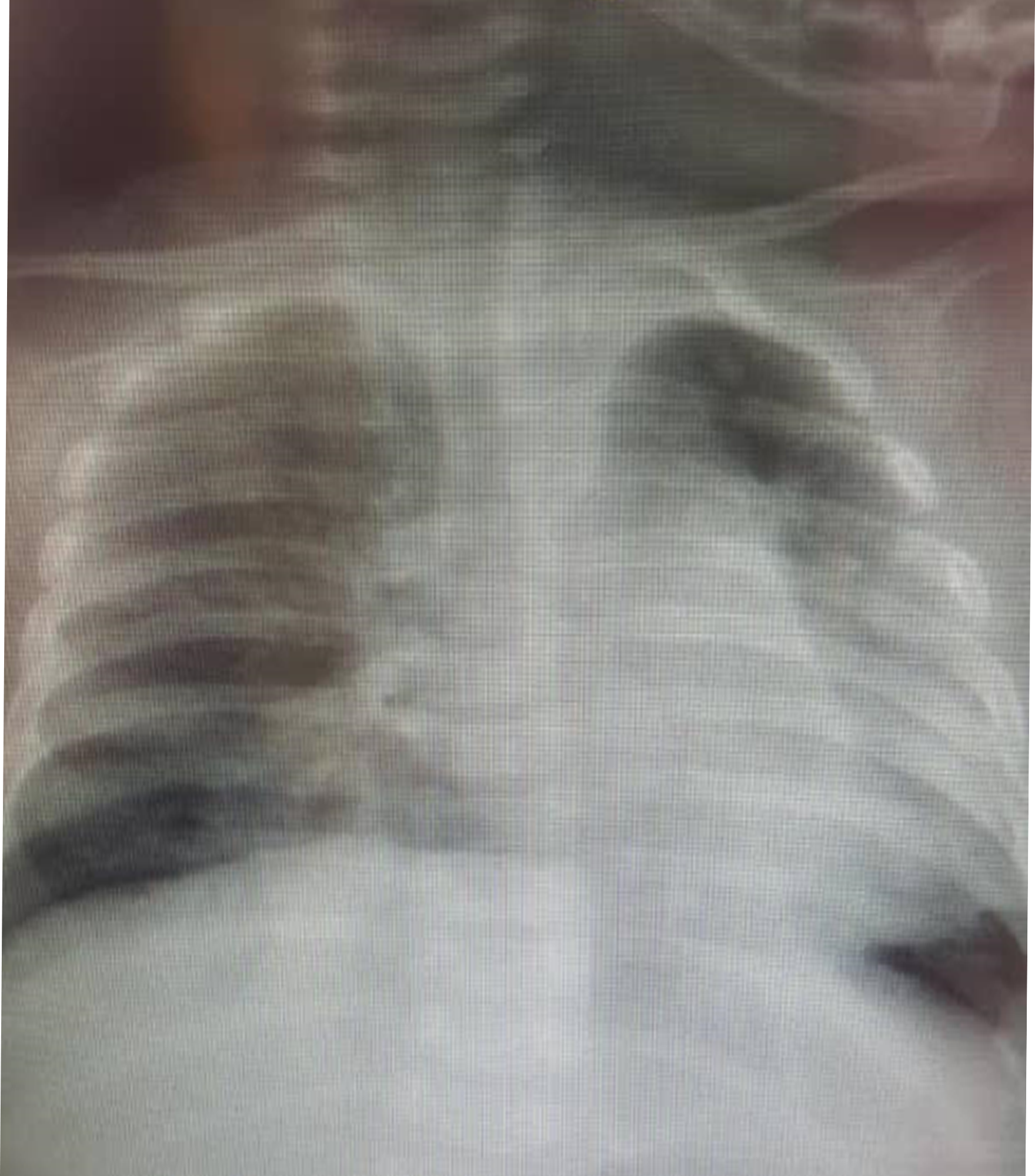


**Figure S9-** Chest computed tomography of P9, 430 months- male with BTK deficiency (X-linked agammaglobulinemia) and COVID-19 infections leading to the recovery.


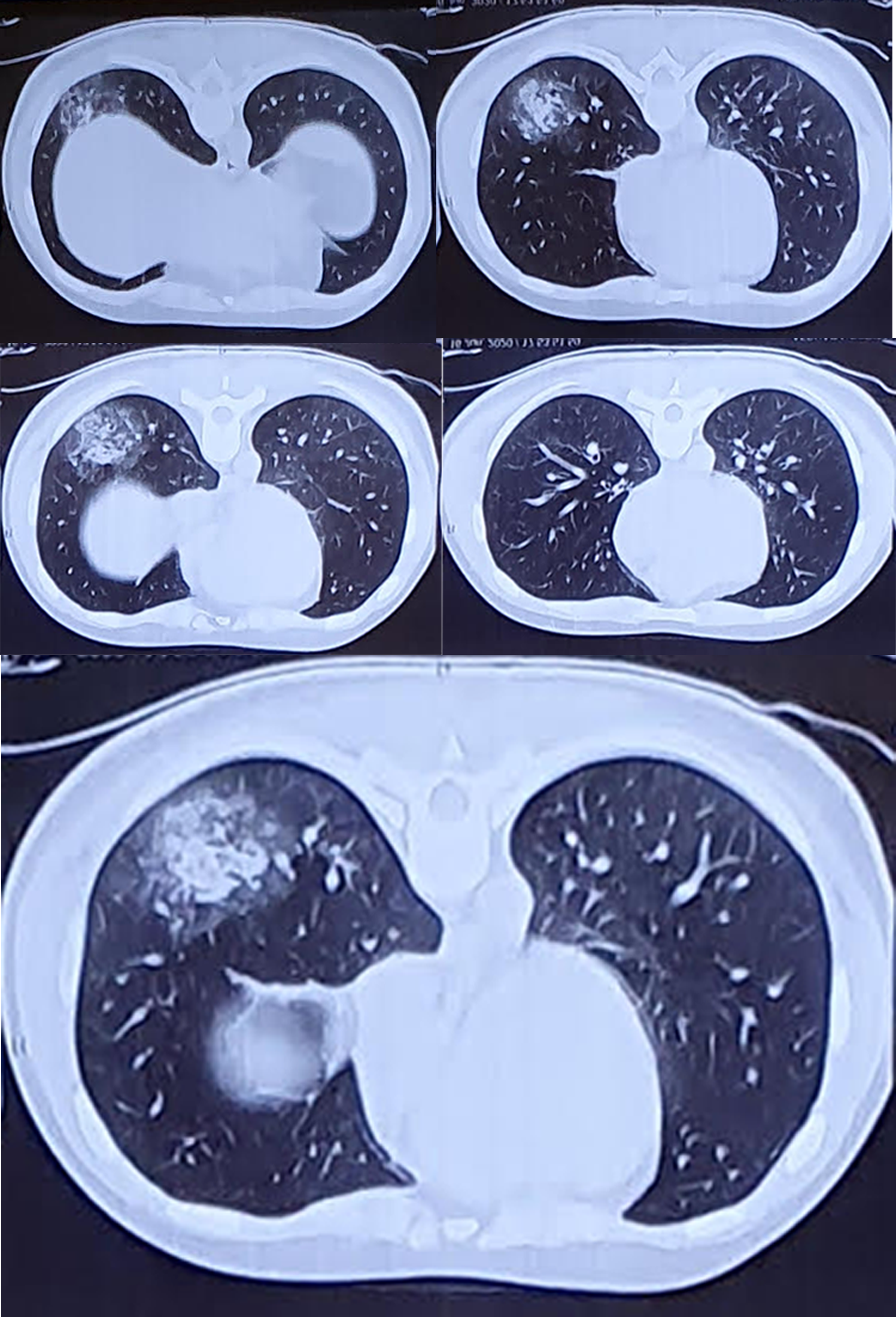


**Figure S10-** Chest computed tomography of P10, 444 months- female with common variable immunodeficiency and COVID-19 infections leading to the recovery.


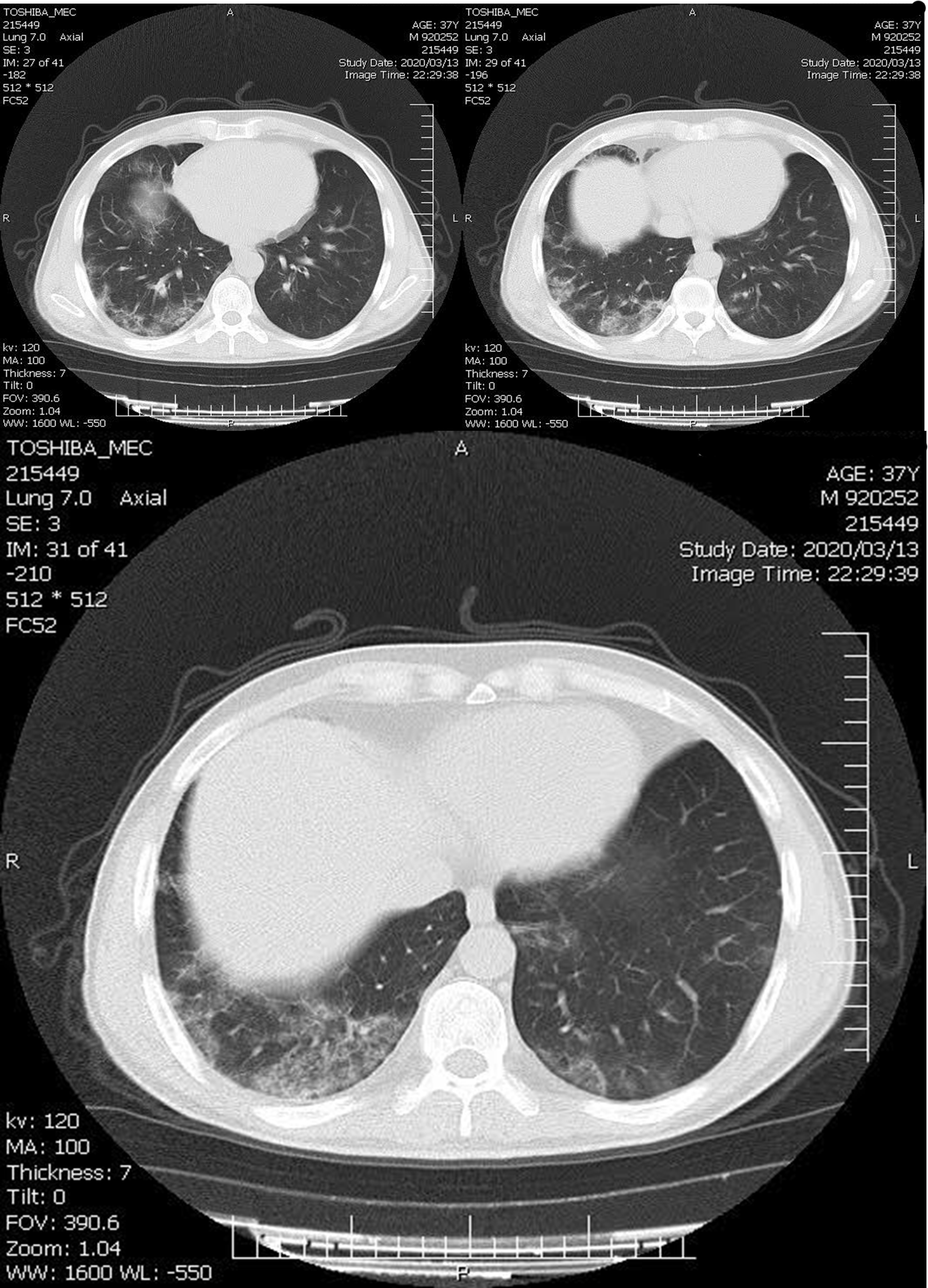


**Figure S11-** Chest computed tomography of P11, 72 months- female with hyper IgM syndrome and COVID-19 infections leading to the recovery.


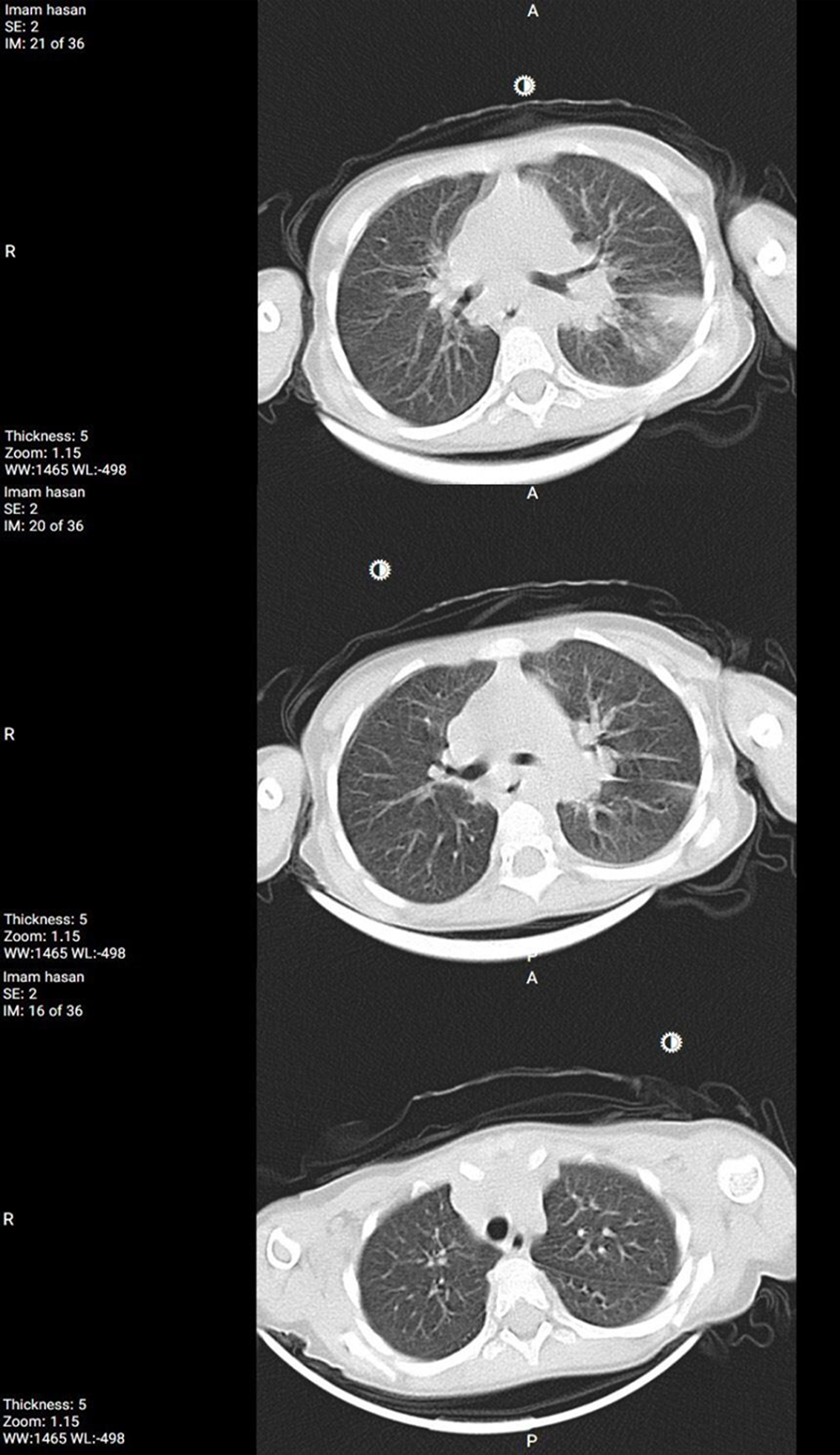


**Figure S12-** Chest X-ray and computed tomography of P12, 96 months- male with selective IgA deficiency and COVID-19 infections leading to the bronchiectasis and collapse consolidations.


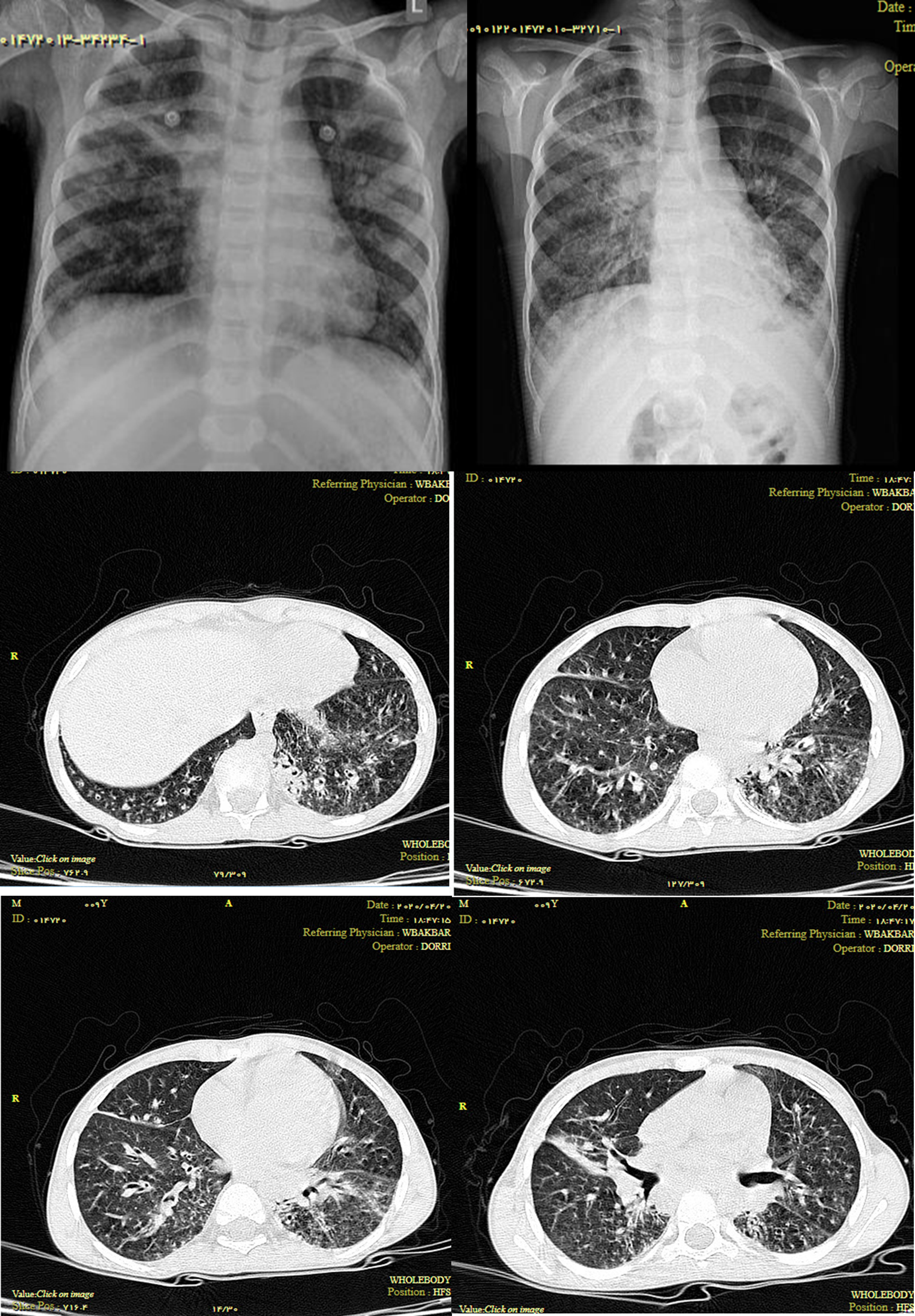


**Figure S13-** Chest computed tomography of P14, 216 months- male with CYBA deficiency and COVID-19 infections leading to the recovery.

**
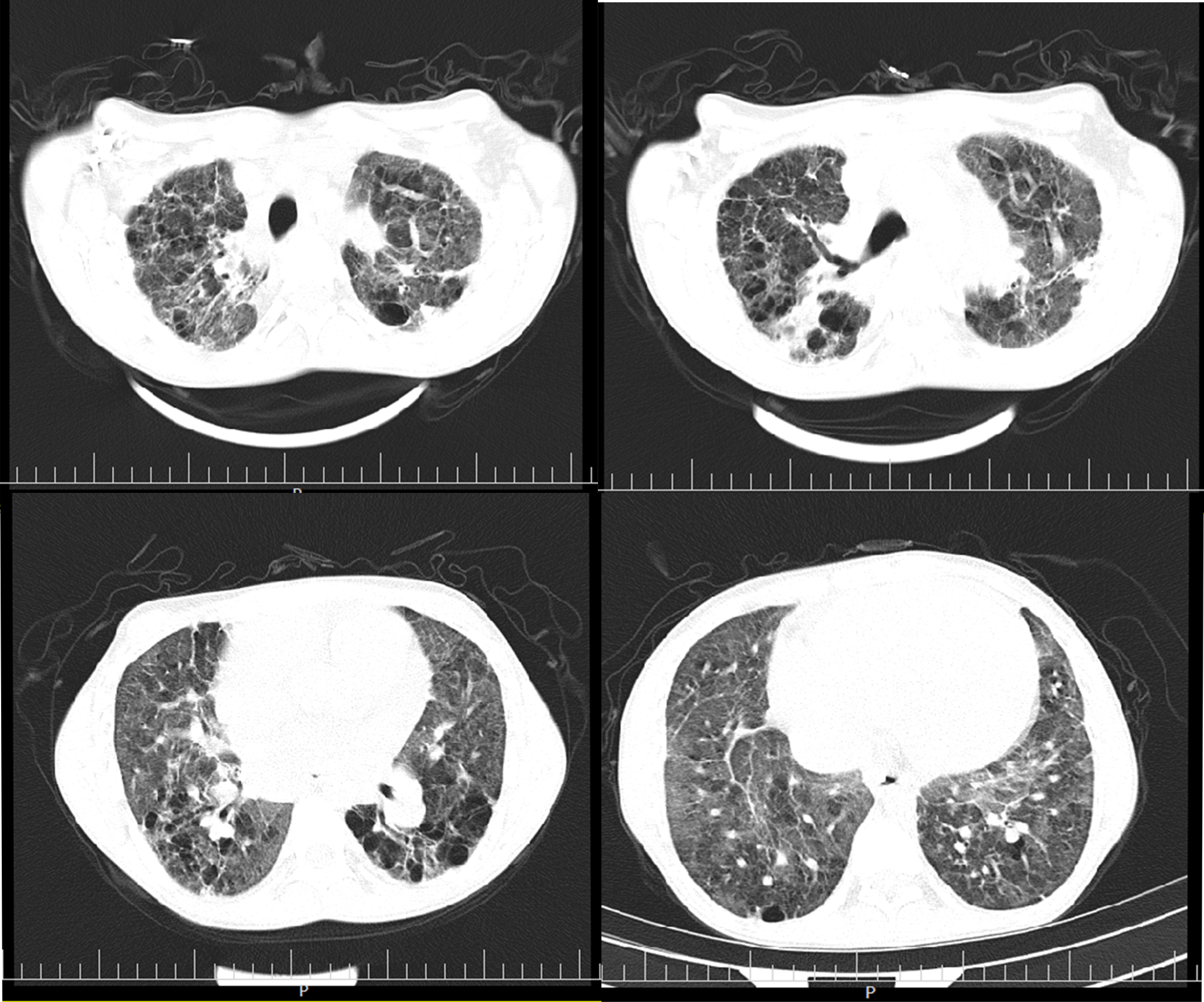
**

**Figure S14-** Chest X-ray and computed tomography of P15, 106 months- female with RAB27A deficiency and COVID-19 infections leading to the death.

**
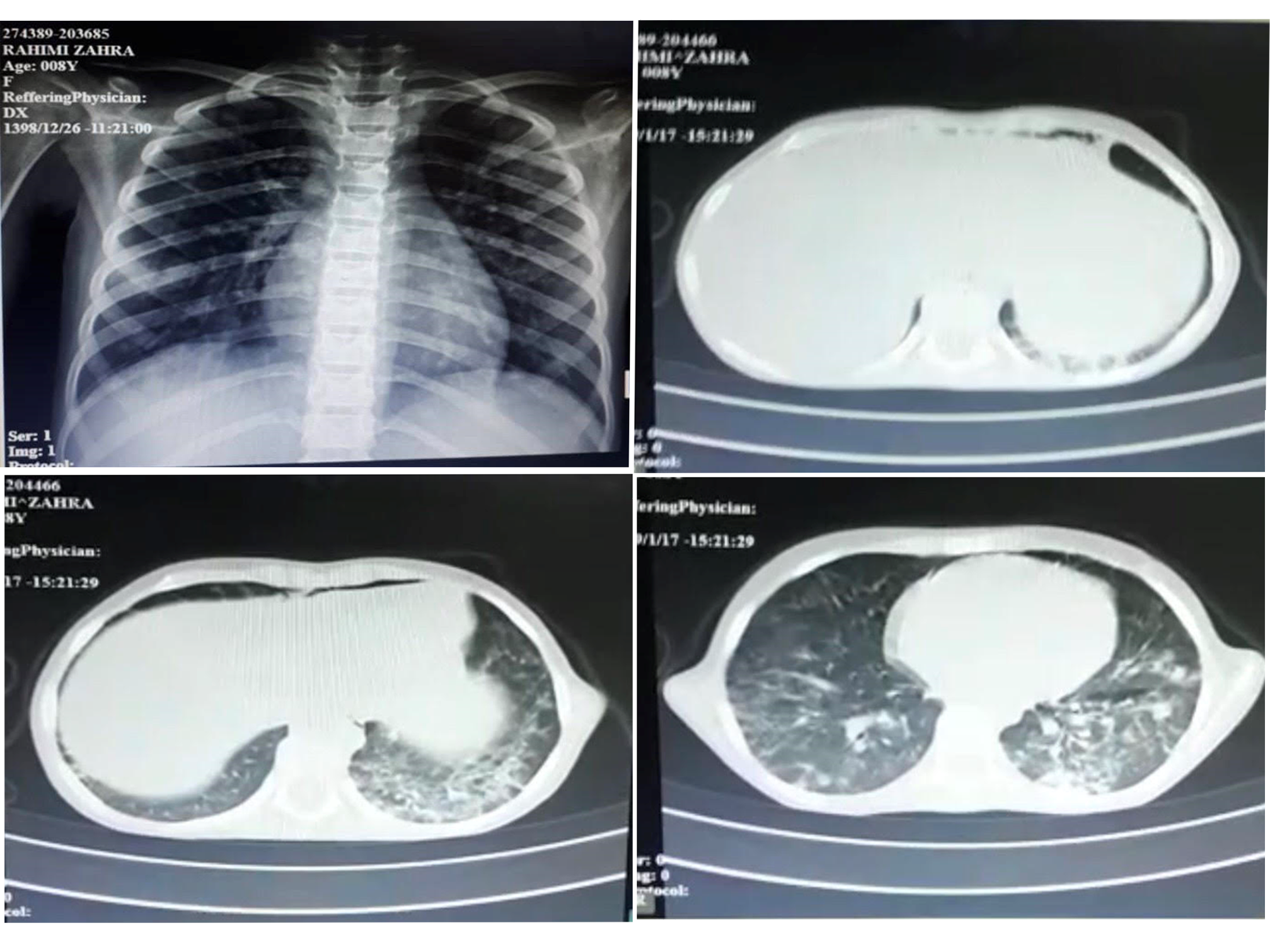
**

**Figure S15-** Chest computed tomography of P16, 372 months- female with CD70 deficiency and COVID-19 infections leading to the recovery.

**
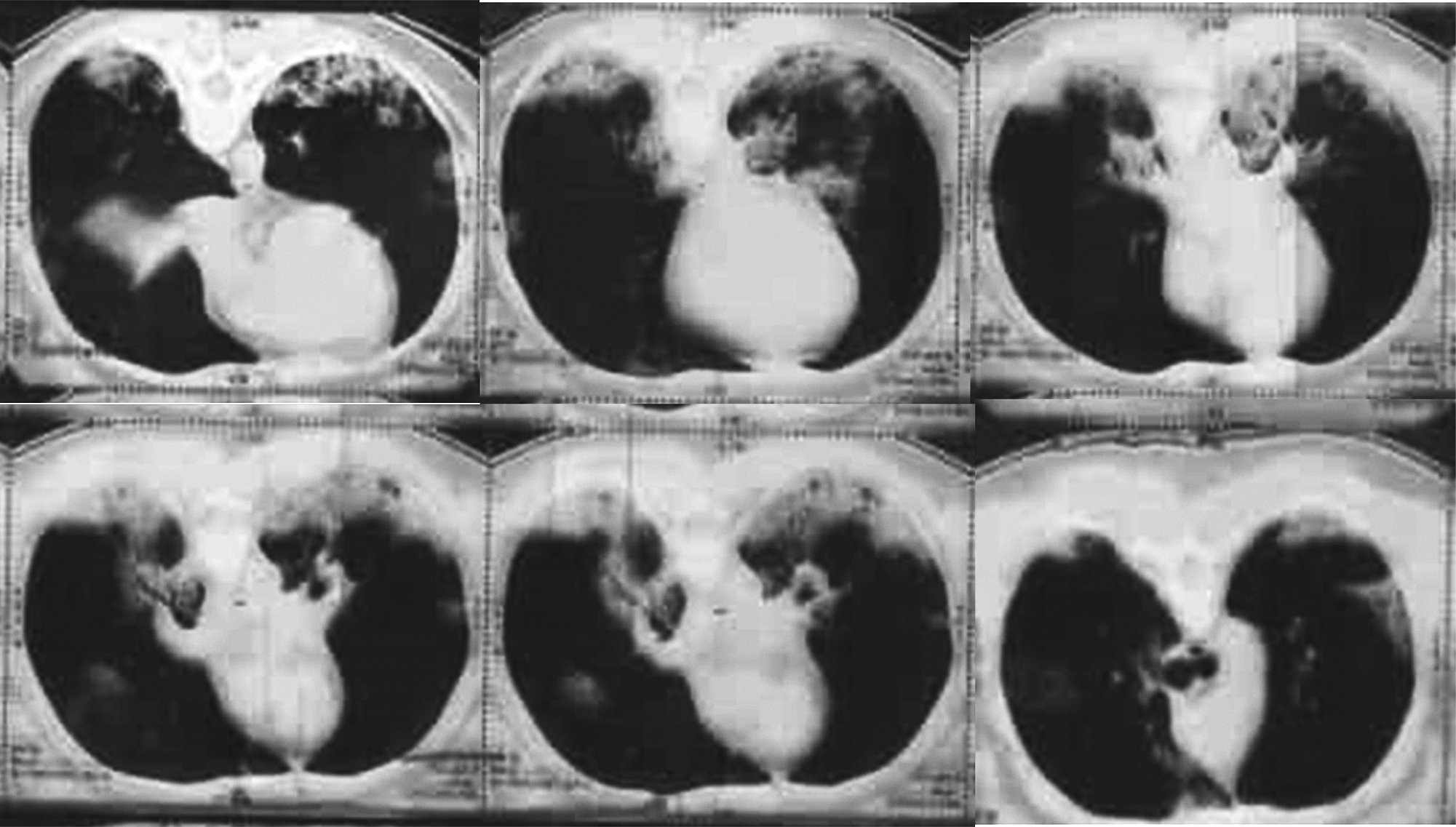
**
